# Supplementary material for: Combining vanadyl sulfate with Newcastle disease virus potentiates rapid innate immune-mediated regression with curative potential in murine cancer models
Source: Mol Ther Oncolytics. 2021 Jan 21;20:306–24. doi: 10.1016/j.omto.2021.01.009 (PMC7868934; doi:10.1016/j.omto.2021.01.009)
Supplement: Document S1. Figures S1–S9 [file mmc1.pdf]

OMTO, Volume *20*

Supplemental Information

Combining vanadyl sulfate with Newcastle disease virus potentiates rapid innate immune-mediated regression with curative potential in murine cancer models

Thomas M. McAusland, Jacob P. van Vloten, Lisa A. Santry, Matthew M. Guilleman, Amira D. Rghei, Edgar M. Ferreira, Joelle C. Ingrao, Rozanne Arulanandam, Pierre P. Major, Leonardo Susta, Khalil Karimi, Jean-Simon Diallo, Byram W. Bridle, and Sarah K. Wootton

A

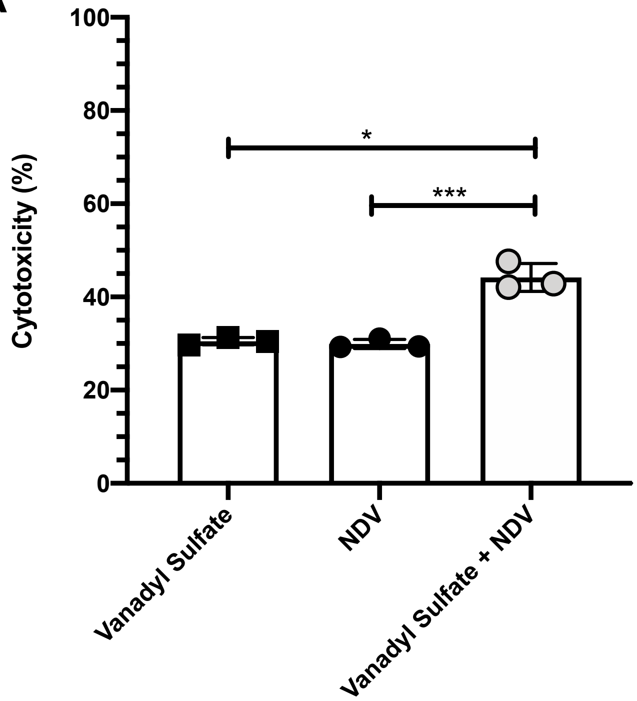

B

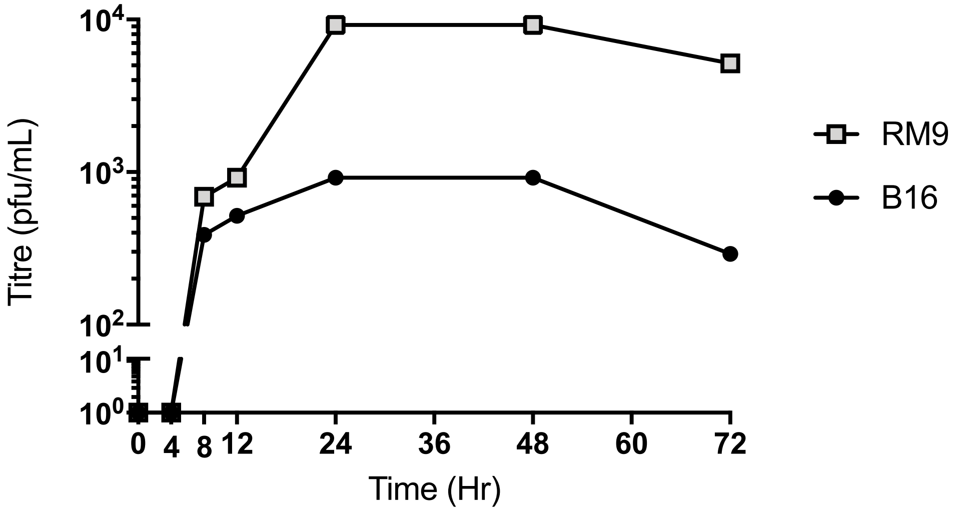

Supplementary Figure 1. (A) Quantification of lactate dehydrogenase (LDH) release from B16-F10 cells treated with NDV, vanadyl sulfate (VS) or a combination of NDV + VS. B16-F10 cells were plated in a 96 well plate at a density of 1×10^4 cells per well. NDV was administered at an MOI of 0.01 and vanadyl sulfate at a concentration of 50 µM. 48 hours after administration of therapies, cell death was quantified by measuring the amount of LDH released from dying cells using the Pierce LDH cytotoxicity assay kit (cat no. 88953 ThermoFisher). Cytotoxicity was measured by subtracting LDH content in remaining viable cells from total LDH in untreated controls. Significance was determined using a one-way ANOVA test (*$p<0.05$, ***$p<0.001$). **(B)** Growth curve of NDV-GFP-F3aa-L289A in murine cancer cell lines. NDV was administered at an MOI of 0.5 to B16-F10 or RM9 cancer cells and supernatant was collected at 0, 4, 8, 12, 24, 48, and 72 hours post infection and applied to newly seeded chicken DF-1 cells to determine titre in pfu/mL. Titers represent an average of triplicates for each time point of each cell line.

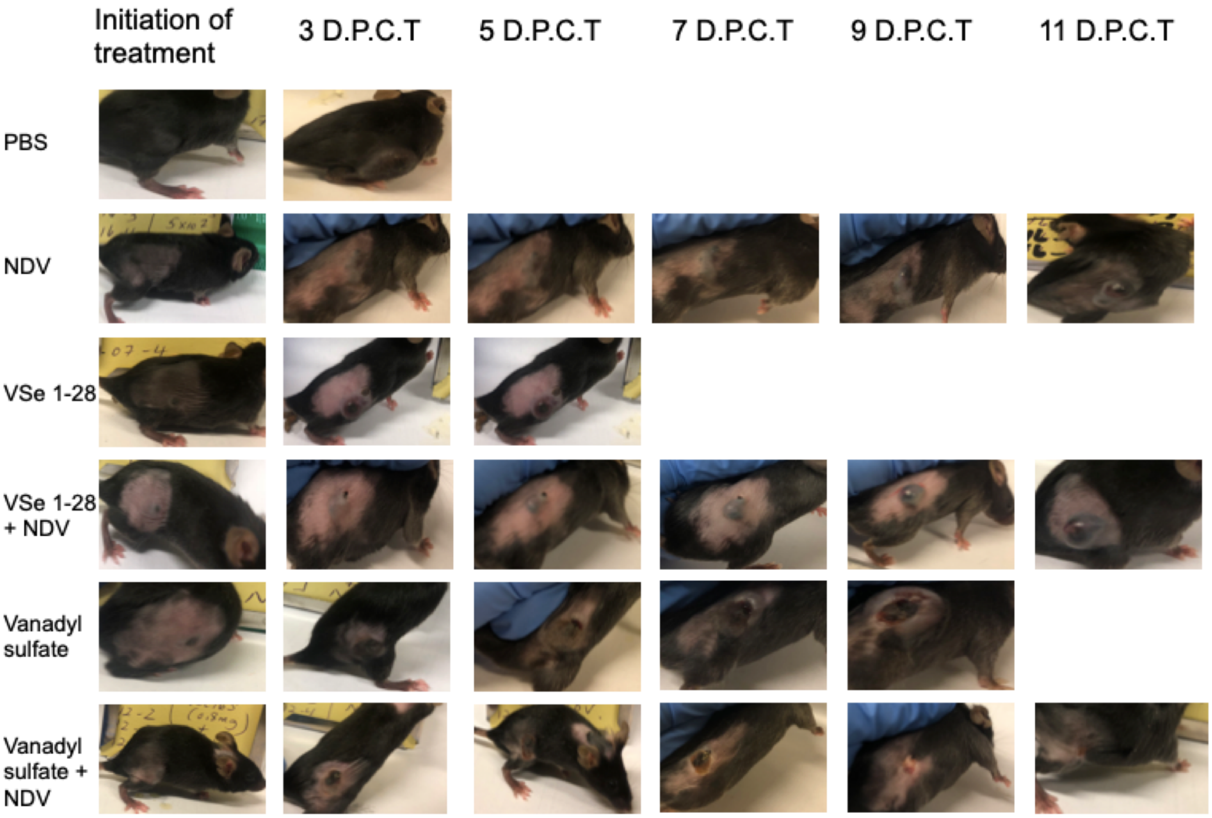

Supplementary Figure 2. Effect of combination therapies on survival in the B16-F10 murine melanoma model. B16-F10 cells (5x10^5 cells) were implanted intradermally and when tumors reached 5 mm in any direction, therapies (phosphate buffered saline (PBS), vanadyl sulfate, VSe 1-28, NDV, or a combination of drug plus NDV) were administered intratumorally every other day for a total of three treatments and tumor volume monitored,. Vanadyl sulfate (40 mg/kg) or VSe1-28 (40 mg/kg) were administered in a volume of 20 uL followed 4 hours later with an intratumoral injection of 5x107 pfu/mL NDV. A representative mouse from each treatment group was imaged starting from the initiation of treatment and varying days post cessation of treatment (D.P.C.T), lack of figure indicates endpoint reached.

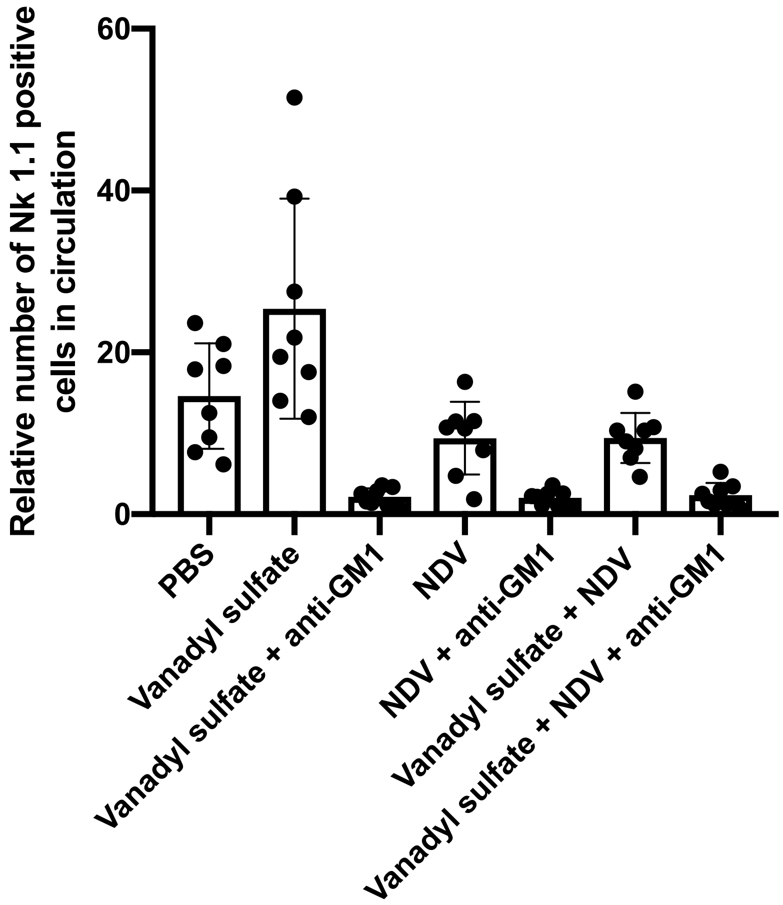

Supplementary Figure 3. Confirmation of NK cell depletion of mice used in vanadyl sulfate plus NDV and NK depletion survival. Blood was drawn retro-orbitally from mice 36 hours after a single treatment of PBS, vanadyl sulfate, NDV, or vanadyl sulfate plus NDV, or the same treatments used in combination with anti-Asialo-GM1 antibody. Blood was then processed using the following antibodies for flow cytometric analysis: BV 421anti-mouse CD3 (BioLegend, cat no. 100336, clone 145-2C11, San Diego, CA), FITC anti-mouse CD69 (BD Bioscience, cat no. 553236, clone H1.2F3, San Jose, CA), APC anti-mouse NK1.1 (BD Bioscience, cat no. 550627, clone PK136, San Jose, CA), and Fc Block-CD16/32 (BioLegend, cat no. 101320, clone 93, San Diego, CA). Cells were isolated from blood, and processed for surface labelling with antibodies against CD3, and NK 1.1, in a single staining panel, followed by the application of fixable viability dye Zombie NIR (BioLegend, San Diego, CA) to label dead cells, followed by fixation with IC fixation buffer (eBioscience, cat no. 00-8222-49, San Diego, CA). Data were acquired using a FACS Canto II flow cytometer with FACSDiva version 8.0 software (BD Biosciences, San Jose, CA) and analyzed using FlowJo version 10.6.2 software (BD Biosciences, San Jose, CA).

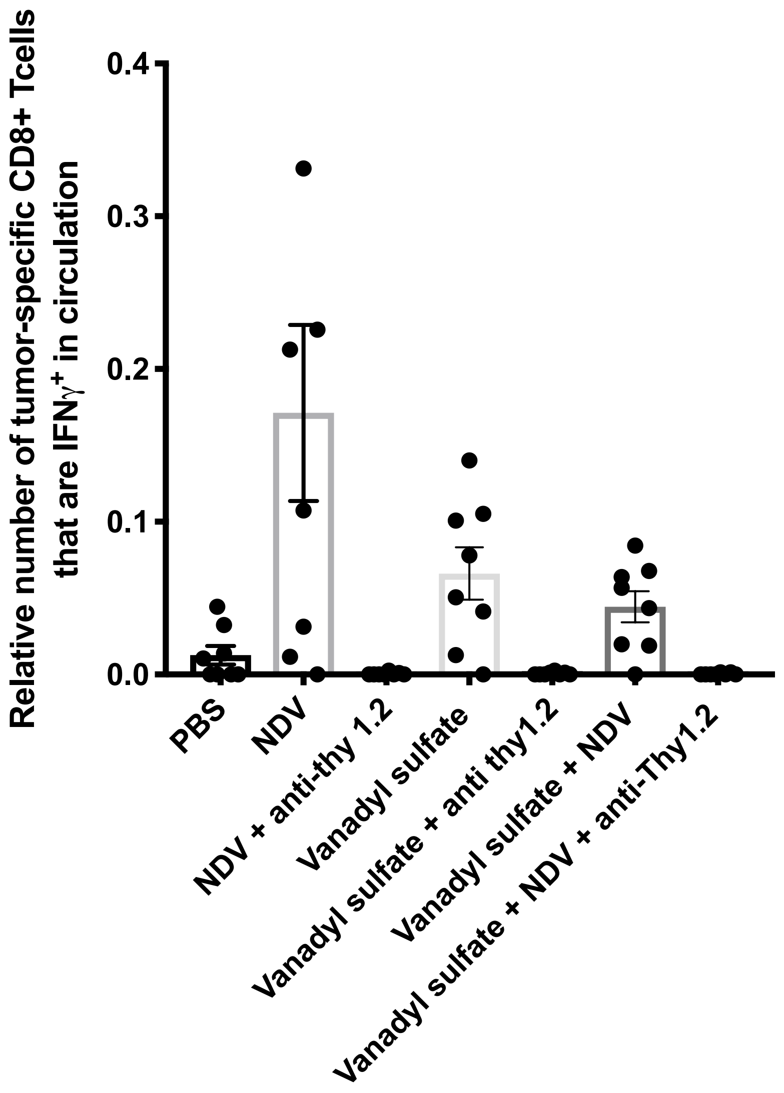

Supplementary Figure 4. Confirmation of T-cell depletion of mice used in vanadyl sulfate plus NDV and T-cell depletion survival. B16-F10 cells seeded at a density of $1x10^5$ cells per well in a round bottom 96-well plate was stimulated with 50 units of murine IFN-γ recombinant protein (eBioscience, cat no. 14-8311-63, San Diego, CA) for 48 hours. Blood was drawn 10 days after the first dose of PBS, vanadyl sulfate (40 mg/kg), NDV ($5x10^7$ pfu/mL), or a combination of vanadyl sulfate plus NDV, or the same treatments used in combination with anti-mouse Thy1.2 (CD90.2; cat no. BE0076, clone 30H12; Bioxcell, Lebanon, NH) antibody and applied to IFN-γ -stimulated B16-F10. Cells were then treated with Fc Block-CD16/32 (BioLegend, cat no. 101320, clone 93, San Diego, CA) , stained and analyzed by flow cytometry after surface staining with antibodies against CD3, FITC anti-mouse CD4 (eBioscience, cat no. 11-0043-85, clone RM4-4, San Diego, CA) and BV 510 anti-mouse CD8 (BioLegend, cat no. 100752, clone 53-6.7, San Diego, CA), and intracellular staining against IFN-γ. T-cell populations were assessed to confirm complete depletion for survival study. Data were acquired using a FACS Canto II flow cytometer with FACSDiva version 8.0 software (BD Biosciences, San Jose, CA) and analyzed using FlowJo version 10.6.2 software (BD Biosciences, San Jose, CA).

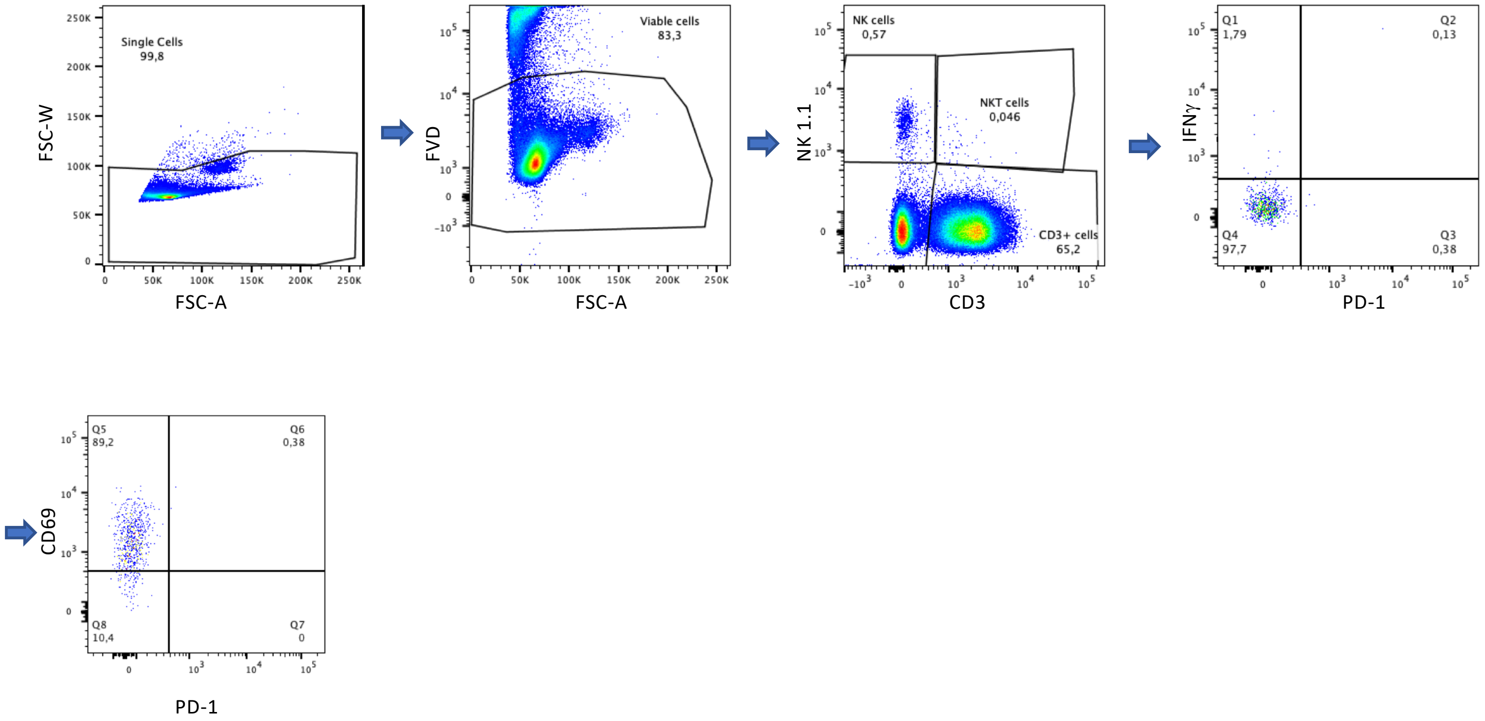

Supplementary Figure 5. Gating strategy to identify IFNγ producing NK cells in the tumor draining lymph node. Gating strategy used to identify single cells, viable cells and NK cells (NK 1.1+ CD3-). Additionally, IFNγ producing NK cells, followed by CD69+ NK cells were identified. Tumor draining lymph nodes were obtained from B16-F10 tumor bearing mice 36 hours after being treated with PBS, vanadyl sulfate, NDV, or a combination of vanadyl sulfate and NDV. Data were acquired using a FACS Canto II flow cytometer with FACSDiva version 8.0 software (BD Biosciences, San Jose, CA) and analyzed using FlowJo version 10.6.2 software (BD Biosciences, San Jose, CA).

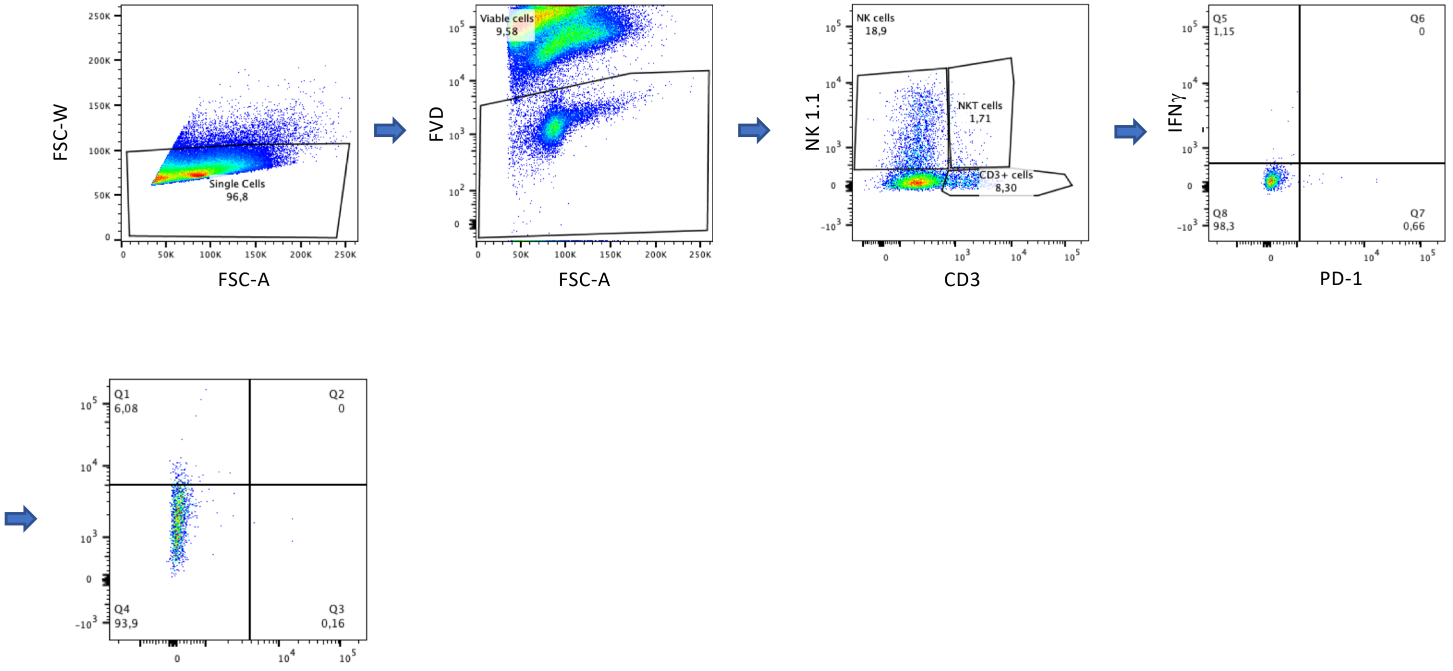

Supplementary Figure 6. Gating strategy to identify IFNγ producing NK cells in the tumor micro-environment. Gating strategy used to identify single cells, viable cells and NK cells (NK 1.1^{+} CD3^{-}). Additionally, IFNγ producing NK cells, followed by CD69+ NK cells were identified. Tumours were obtained from B16-F10 tumor bearing mice 36 hours after being treated with PBS, vanadyl sulfate, NDV, or a combination of vanadyl sulfate and NDV. Data were acquired using a FACS Canto II flow cytometer with FACSDiva version 8.0 software (BD Biosciences, San Jose, CA) and analyzed using FlowJo version 10.6.2 software (BD Biosciences, San Jose, CA).

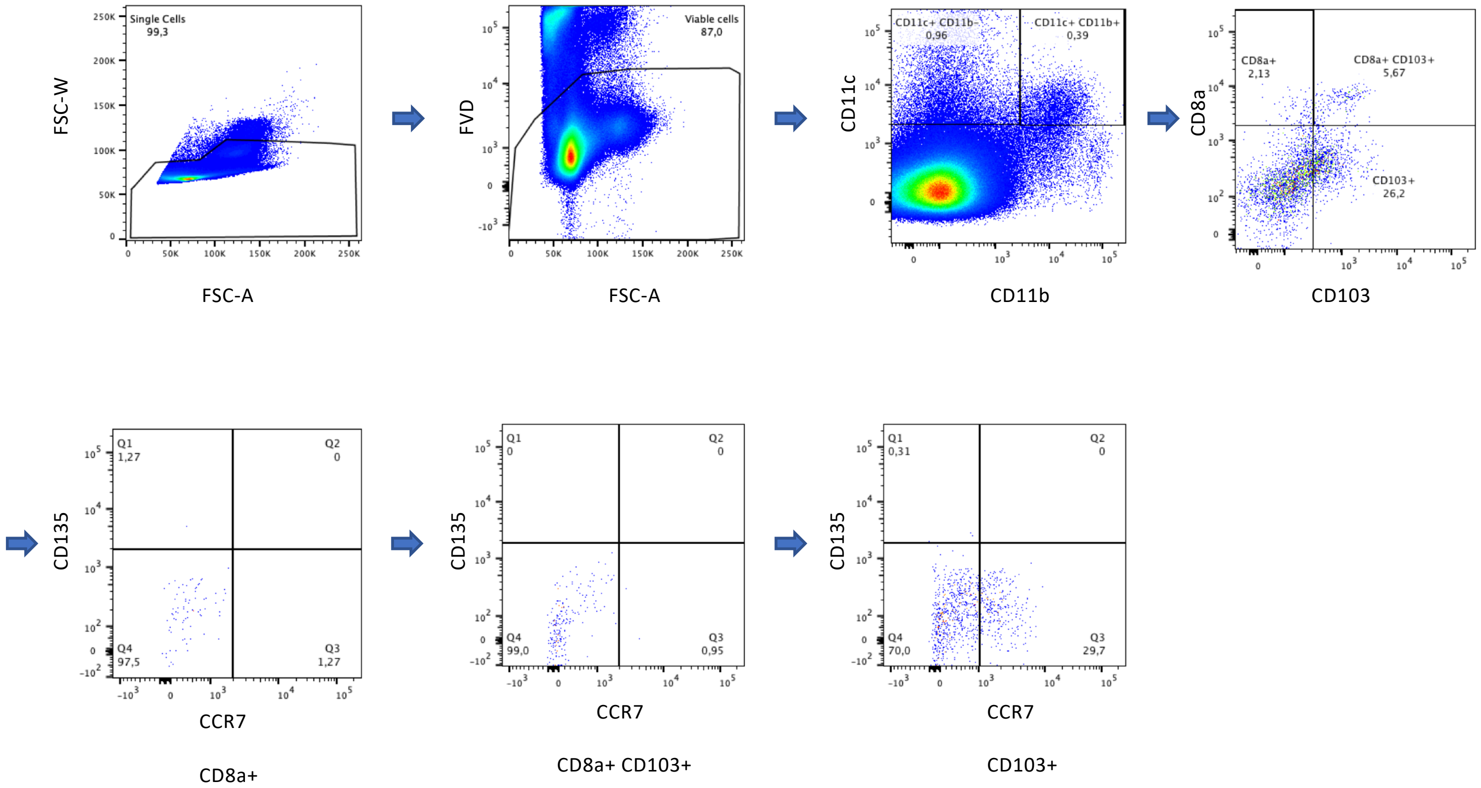

Supplementary Figure 7. Gating strategy to identify classical dendritic cell (cDC) populations in the tumor draining lymph node. Gating strategy used to identify single cells, viable cells, and then divided cells into CD11b+ and CD11c+ cells. Cells were then further divided using CD8a and CD103 staining, which identified single and double positives. CD8a+, CD103+, or CD8a and CD103+ populations were then analyzed for CD135 and CCR7. Tumours were obtained from B16-F10 tumor bearing mice 36 hours after being treated with PBS, vanadyl sulfate, NDV, or a combination of vanadyl sulfate and NDV. Data were acquired using a FACS Canto II flow cytometer with FACSDiva version 8.0 software (BD Biosciences, San Jose, CA) and analyzed using FlowJo version 10.6.2 software (BD Biosciences, San Jose, CA).

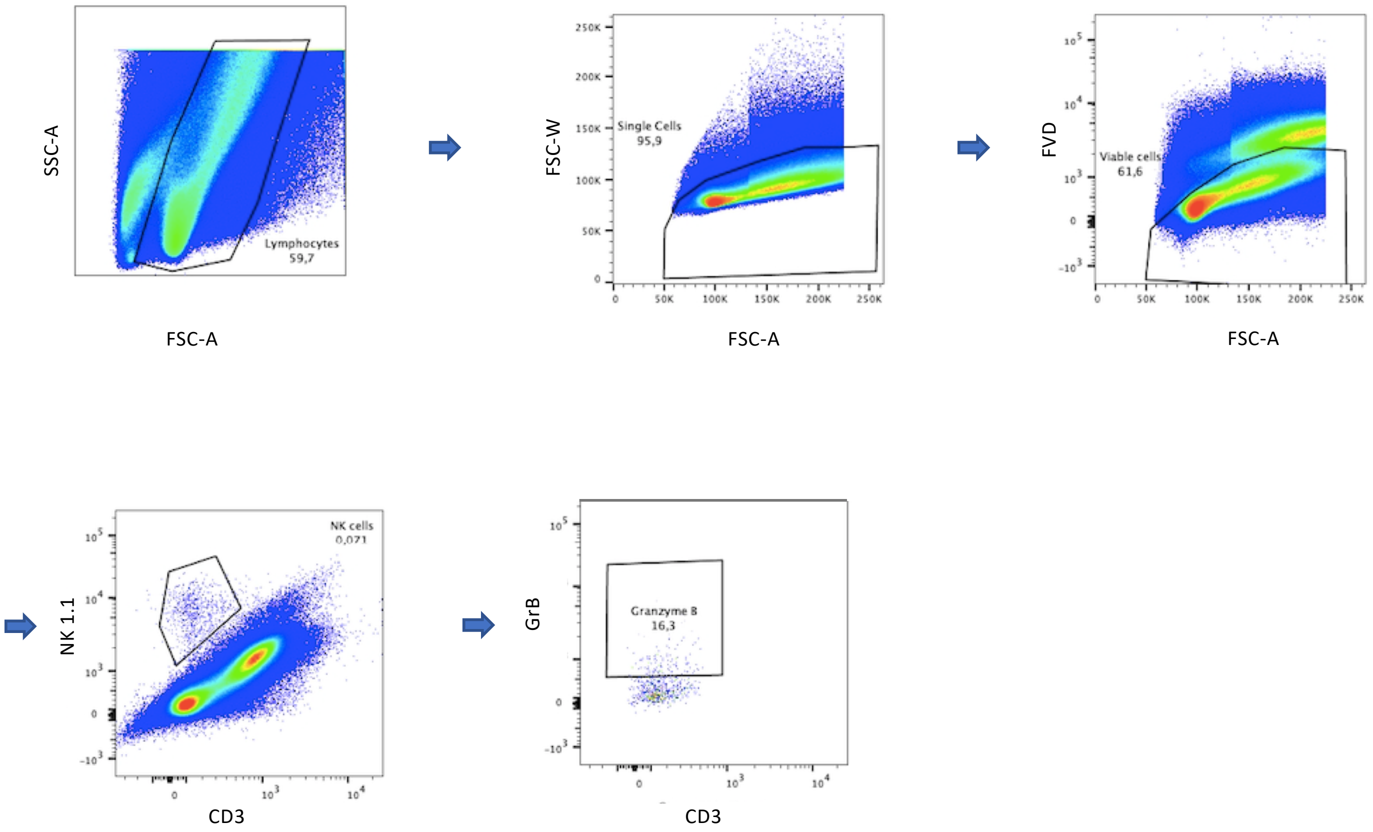

Supplementary Figure 8. Gating strategy to identify Granzyme-B producing NK cells at an acute time point in the tumor micro-environment. Gating strategy used to identify lymphocytes, single cells, viable cells, and NK 1.1+ CD3- cells, as NK cells. NK cells were then analyzed for Granzyme-B levels. Tumours were obtained from B16-F10 tumor bearing mice 24 hours after being treated with PBS, vanadyl sulfate, NDV, or a combination of vanadyl sulfate and NDV. Data were acquired using a FACS Canto II flow cytometer with FACSDiva version 8.0 software (BD Biosciences, San Jose, CA) and analyzed using FlowJo version 10.6.2 software (BD Biosciences, San Jose, CA).

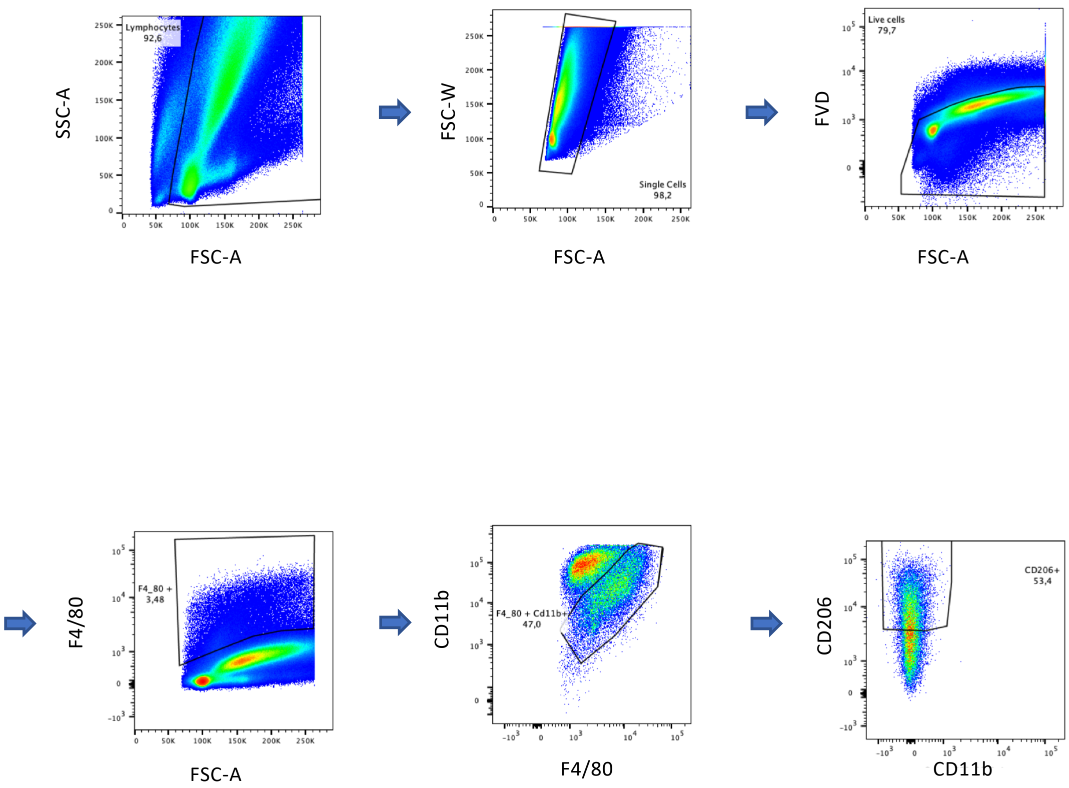

Supplementary Figure 9. Gating strategy to identify CD206 positive macrophage populations. Gating strategy used to identify lymphocytes, single cells, viable cells followed by F4/80+ cells. Cells were then identified as CD11b+ and lastly CD206+. Tumours were obtained from B16-F10 tumor bearing mice 24 hours after being treated with PBS, vanadyl sulfate, NDV, or a combination of vanadyl sulfate and NDV. Data were acquired using a FACS Canto II flow cytometer with FACSDiva version 8.0 software (BD Biosciences, San Jose, CA) and analyzed using FlowJo version 10.6.2 software (BD Biosciences, San Jose, CA).
