# Supplementary material for: Combining vanadyl sulfate with Newcastle disease virus potentiates rapid innate immune-mediated regression with curative potential in murine cancer models
Source: Mol Ther Oncolytics. 2021 Jan 21;20:306–24. doi: 10.1016/j.omto.2021.01.009 (PMC7868934; doi:10.1016/j.omto.2021.01.009)
Supplement: Document S2. Article plus supplemental information [file mmc2.pdf]

Molecular Therapy
Oncolytics
Original Article

Combining vanadyl sulfate with Newcastle disease virus potentiates rapid innate immune-mediated regression with curative potential in murine cancer models

Thomas M. McAusland,[1] Jacob P. van Vloten,[1] Lisa A. Santry,[1] Matthew M. Guilleman,[1] Amira D. Rghei,[1] Edgar M. Ferreira,[1] Joelle C. Ingrao,[1] Rozanne Arulanandam,[2,3] Pierre P. Major,[4] Leonardo Susta,[1] Khalil Karimi,[1] Jean-Simon Diallo,[2,3] Byram W. Bridle,[1] and Sarah K. Wootton[1]

[1]Department of Pathobiology, University of Guelph, Guelph, ON N1G 2WI, Canada; [2]Center for Innovative Cancer Research, Ottawa Hospital Research Institute, Ottawa, ON, Canada; [3]Department of Biochemistry, Microbiology, and Immunology, Faculty of Medicine, University of Ottawa, Ottawa, ON, Canada; [4]Juravinski Cancer Centre, 699 Concession Street, Hamilton, ON L8V 5C2, Canada

The avian paramyxovirus, Newcastle disease virus (NDV), is a promising oncolytic agent that has been shown to be safe and effective in a variety of pre-clinical cancer models and human clinical trials. NDV preferentially replicates in tumor cells due to signaling defects in apoptotic and antiviral pathways acquired during the transformation process and is a potent immunostimulatory agent. However, when used as a monotherapy NDV lacks the ability to consistently generate durable remissions. Here we investigate the use of viral sensitizer-mediated combination therapy to enhance the anti-neoplastic efficacy of NDV. Intratumoral injection of vanadyl sulfate, a pan-inhibitor of protein tyrosine phosphatases, in combination with NDV significantly increased the number and activation status of natural killer (NK) cells in the tumor microenvironment, concomitant with increased expression of interferon-β, granulocyte-macrophage colony-stimulating factor, and monocyte chemoattractant protein-1, leading to rapid tumor regression and long-term cures in mice bearing syngeneic B16-F10 melanomas. The anti-tumor efficacy of this combination therapy was abrogated when NK cells were depleted and when interferon-β expression was transiently suppressed. Tumor-specific $CD8^{+}$ T cell responses were not detected, nor were mice whose tumors regressed protected from re-challenge. This suggested efficacy of the combination therapy predominantly relied on the innate immune system. Importantly, efficacy was not limited to melanoma; it was also demonstrated in a murine prostate cancer model. Taken together, these results suggest that combining NDV with vanadyl sulfate potentiates an innate immune response that can potentiate rapid clearance of tumors, with type I interferon signaling and NK cells being important mechanisms of action.

INTRODUCTION

Cancer immunotherapy represents a novel approach to treat malignancies, whereby the patient's suppressed immune system is revived so that it is again capable of launching sustained attacks against tumor cells.[1] Oncolytic viruses (OVs), such as Newcastle disease virus (NDV), reovirus, herpes simplex virus (HSV)-1, and vaccinia virus, are important immune-potentiating agents in the cancer immunotherapy armamentarium, as these viruses preferentially replicate in tumor cells, resulting in inflammatory responses that lead to activation of innate and adaptive immune responses.[2] NDV is an attractive candidate for oncolytic immunotherapy due to its preferential replication in tumor cells possessing defects in their apoptotic and antiviral response pathways and its remarkable safety profile.[3–6]

NDV is a member of the *Orthoavulavirus* genus in the *Paramyxoviridae* family, and field strains are associated with respiratory infections in a range of avian species; however, NDV is not known to cause disease in humans.[7] When used as a monotherapy in pre-clinical models, NDV has been shown to possess a variety of direct and indirect immunostimulatory and anti-tumor properties.[8] Recombinant NDV has been engineered to contain a multibasic cleavage site in the fusion protein (NDV-F3aa[9] or NDV-F3aa[L289A][10]) to increase fusogenicity, as well as to express a variety of therapeutic transgenes, including interleukin (IL)-2,[9] granulocyte-macrophage colony-stimulating factor (GM-CSF),[11] IL-15,[12] immunoglobulins against extradomain B of fibronectin,[13] inducible T cell co-stimulator (ICOS) ligand,[14] cytotoxic T lymphocyte antigen (CTLA)-4,[15] and programmed death protein (PD)-1/PD ligand-1,[16] to further enhance its anti-neoplastic capabilities. While vectorization of these transgenes has improved the potency of NDV, complete cures remain elusive. The use of NDV in combination with systemic immune checkpoint antibodies

Received 8 January 2021; accepted 16 January 2021;
https://doi.org/10.1016/j.omto.2021.01.009.

Correspondence: Sarah K. Wootton, Department of Pathobiology, University of Guelph, Guelph, ON N1G 2WI, Canada.
E-mail: kwootton@uoguelph.ca

This is an open access article under the CC BY license (http://creativecommons.org/licenses/by/4.0/).

(e.g., anti-CTLA-4 and anti-PD-1) has been shown to significantly enhance survival in mouse models of melanoma, prostate, and bladder cancers.[15,17,18] The use of such systemic antibodies has proven to be efficacious; however, toxicities and off-target effects remain a concern.[19] The use of complementary agents that are less toxic may prove to be a comparable, if not more efficacious, approach.

The term “viral sensitizer” or VSe was first coined by Diallo et al.[20] and describes a growing category of small-molecule pharmacological agents that have been shown to enhance viral oncolysis. While the mechanism of many VSes remains unknown,[21] some elicit their effects by increasing viral titers by disrupting the interferon (IFN)-induced antiviral response through a variety of targets, including nuclear factor-κB (NF-κB),[20,22] microtubule destabilization,[23] and histone deacetylase (HDAC) inhibition.[24] Drugs such as dimethyl fumarate, HDAC inhibitors, fluphenazine, indirubin, lofepramine, ranolazine, vanadate, and pyrrole derivatives have all been shown to synergize with a range of OVs in various murine cancer models.[21,22,24–26]

Vanadium is a naturally occurring oxo-metalate that has previously been utilized in phase I/II clinical trials for treatment of diabetes for its insulin-like effects, specifically its ability to stimulate glucose, glycogen synthesis, and inhibition of gluconeogenesis in hepatic cells.[27–30] Vanadyl sulfate, an oxidative form of vanadium, is a commonly used body-building supplement. Recent research has suggested that, in addition to their insulin-mimetic properties, vanadium compounds possess anti-neoplastic properties due to their activity as pan tyrosine phosphatase inhibitors and their ability to stimulate the immune system through the induction of pro-inflammatory cytokines, which lead to an influx of granulocytes.[31,32] In some instances, vanadium compounds have been shown to induce apoptosis through the generation of reactive oxygen species and to promote cell cycle arrest by counteracting mitogen-activated protein kinase signaling and strongly inducing p21Cip1 expression and retinoblastoma hypophosphorylation;[33] however, this was not the case for A549 cells.[34] In the context of cancer cells, vanadate significantly decreases the antiviral effects of type I IFN, while increasing the production of pro-inflammatory cytokines and chemokines. Combination of vanadate and oncolytic vesicular stomatitis virus (VSVΔ51) was shown to increase viral spread and enhance survival in several immunocompetent mouse tumor models, with comparatively reduced anti-tumor effects in T cell-deficient mice.[21]

In contrast with vanadium compounds, synthetic agents such as VSe 1 and its pyrrole derivative VSe 1-28 elicit a more focused effect by transiently suppressing the type I IFN response, specifically through transcriptional repression of type I IFN-stimulated genes (ISGs), ultimately leading to increased viral replication as demonstrated in studies using VSVΔ51 and other IFN-sensitive viruses, such as ICP0 null HSV-1.[35]

In this study, we compared the effects of the “pro-inflammatory” VSe vanadyl sulfate to that of VSe 1-28 on the efficacy of oncolytic NDV-F3aa(L289A) *in vitro* and in immunocompetent murine cancer models. We demonstrated that combination vanadyl sulfate-NDV treatment led to rapid tumor regression and complete cures in a B16-F10 melanoma model. Efficacy was not limited to the route of administration or cancer type. We also addressed the contributions of various cells of the innate and adaptive immune system and identified a signaling pathway that was required to elicit these profound anti-tumor effects.

RESULTS

VSes act in conjunction with NDV to reduce metabolic activity and modulate IFN-β expression in B16-F10 murine melanoma cells

To determine whether the VSes vanadyl sulfate and VSe 1-28 enhance the ability of NDVF3aa (L289A) carrying a transgene encoding full-length enhanced green fluorescent protein (GFP; this virus herein referred to as NDV) to reduce B16-F10 cell viability *in vitro*, two-fold dilutions of VSe were applied to B16-F10 cells, and then 4 h later the cells were treated with NDV at a multiplicity of infection (MOI) of 0.01. After 48 h, metabolic activity, which is a surrogate for cell viability, was quantified using a resazurin assay[36] and compared to control cells that were untreated or treated with a VSe or NDV as single agents. Combinations were deemed to have enhanced oncolysis if cell viability was significantly lower than both NDV and the VSe when used as monotherapies. The combination of 50 μM vanadyl sulfate plus NDV significantly reduced the metabolic activity of B16-F10 cells (Figure 1A) and significantly increased cell lysis as measured by release of lactate dehydrogenase (Figure S1) when compared to each of the monotherapies on their own. Similarly, the combination of 0.39 μM or 0.79 μM of VSe 1-28 and NDV significantly reduced metabolic activity compared to NDV or VSe 1-28 alone (Figure 1B).

To determine whether combining VSes with NDV has the potential to increase therapeutic transgene expression, the amount of NDV-mediated expression of GFP in B16-F10 cells treated with vanadyl sulfate or VSe 1-28 was measured using a plate reader and compared to expression mediated by the parental NDV that lacked a transgene. As shown in Figures 1C and 1D, pre-treating B16-F10 cells with vanadyl sulfate or VSe 1-28 4 h prior to infection with recombinant NDV expressing GFP did not significantly increase overall expression of GFP.

The fact that the combination therapy did not enhance NDV-mediated transgene expression suggested that replication of the virus may not have been potentiated. To investigate this, B16-F10 cells were pre-treated with vanadyl sulfate or VSe 1-28 for 4 h before being treated with NDV at a MOI of 0.01. After 48 h, the supernatants were collected and virus titered on DF-1 cells by 50% tissue culture infective dose ($TCID_{50}$) assay. No significant increases in NDV titers were observed with either treatment (Figure 1E). Taken together, these data demonstrate that combining low doses of vanadyl sulfate or VSe 1-28 with NDV significantly reduces the viability of B16-F10 tumor cells relative to VSe or NDV alone but does not appear to enhance viral replication or transgene expression in these cells.

Vanadyl sulfate and VSe 1-28 have previously been shown to modulate IFN-β signaling.[21,35] To investigate the impact of VSe plus NDV

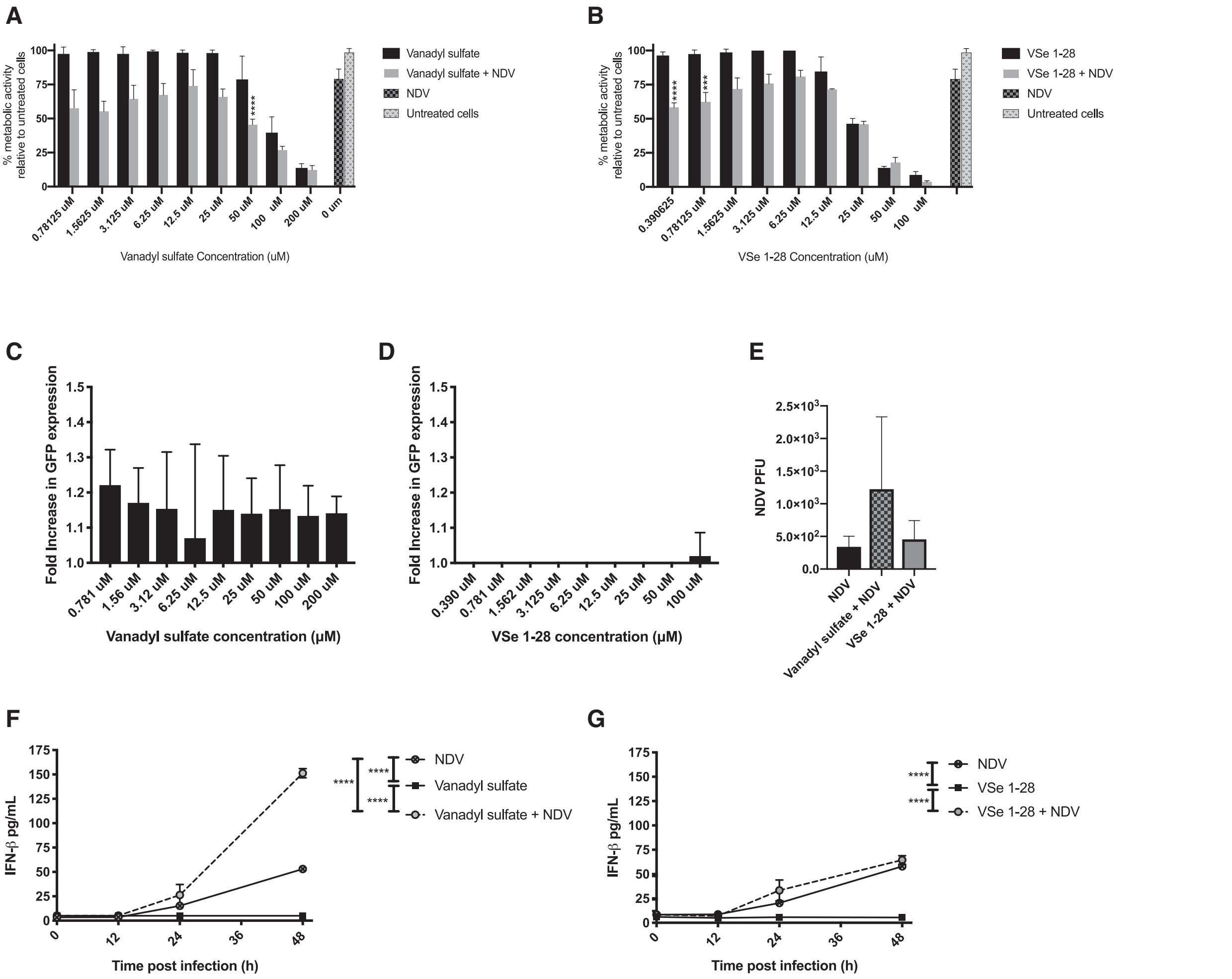

Figure 1. Impact on cell viability, virus production, and interferon (IFN)-β expression in B16-F10 cells treated with VSe 1-28 and vanadyl sulfate either alone or in combination with NDV

(A) Percent metabolic activity of B16-F10 cells treated with vanadyl sulfate, NDV (multiplicity of infection [MOI] = 0.01) or vanadyl sulfate + NDV compared to vehicle treatment alone. (B) Percent metabolic activity of B16-F10 cells treated with VSe 1-28, NDV (MOI = 0.01), or VSe 1-28 + NDV compared to vehicle treatment alone. Data were analyzed by 2-way analysis of variance (ANOVA) using a Tukey's multiple comparison test, comparing the mean of each column with the mean of every other column. Significance was determined when comparing vanadyl sulfate + NDV or VSe 1-28 + NDV to NDV control only if the drug alone did not reduce metabolic activity below 80% (***$p < 0.001$, ****$p < 0.0001$). (C and D) Fold increase in GFP expression from B16-F10 cells treated with vanadyl sulfate (50 μM) (C), or VSe 1-28 (0.390 μM) (D), and then infected with NDV (MOI 0.01) relative to cells infected with NDV-GFP alone. Data were analyzed using a one-way ANOVA. (E) Amount of NDV produced from B16-F10 cells treated with NDV alone (MOI = 0.01) or NDV in combination with either VSe 1-28 (0.390 μM) or vanadyl sulfate (50 μM). The Spearman-Kärber method was used to determine plaque-forming units (PFU)/mL. A one-way ANOVA was used to determine significance (*$p < 0.05$, **$p < 0.01$, ***$p < 0.001$, ****$p < 0.0001$). (F and G) B16-F10 cells were treated with phosphate-buffered saline (PBS), vanadyl sulfate either alone or in combination with NDV (MOI = 0.01) (F) and B16-F10 cells were treated with PBS or VSe 1-28 either alone or in combination with NDV (MOI = 0.01) (G). NDV supernatants were collected at 0, 12, 24, and 48 h post-treatment. Significance was observed when comparing NDV versus VSe 1-28 ($p = 0.003$), NDV versus vanadyl sulfate ($p = 0.0001$), NDV versus vanadyl sulfate + NDV ($p < 0.0001$), VSe 1-28 versus VSe 1-28 + NDV ($p < 0.0001$), VSe 1-28 versus vanadyl sulfate + NDV ($p < 0.0001$), VSe 1-28 + NDV versus vanadyl sulfate ($p < 0.0001$), VSe 1-28 + NDV versus vanadyl sulfate + NDV ($p = 0.0066$), and vanadyl sulfate versus vanadyl sulfate + NDV ($p < 0.0001$). Quantities made relative to cell only control. Significance was determined using a 2-way ANOVA test. Experiments were conducted a minimum of three times.

combination therapy on IFN-β expression, B16-F10 cells were treated with 50 μM of vanadyl sulfate, 0.390 μM of VSe 1-28, NDV (MOI of 0.01), or a combination of NDV plus vanadyl sulfate or VSe 1-28, and the amount of IFN-β produced at 0, 12, 24, and 48 h post-infection was quantified using a commercial enzyme-linked immunosorbent assay (ELISA). As shown in Figure 1F, the combination of vanadyl sulfate plus NDV significantly increased the concentration of IFN-β in the supernatant compared to NDV or vanadyl sulfate alone. While combining VSe 1-28 with NDV led to a significant increase in IFN-β production compared to VSe 1-28 alone, this was not significantly different from NDV alone (Figure 1G). These data demonstrate that vanadyl sulfate plus NDV

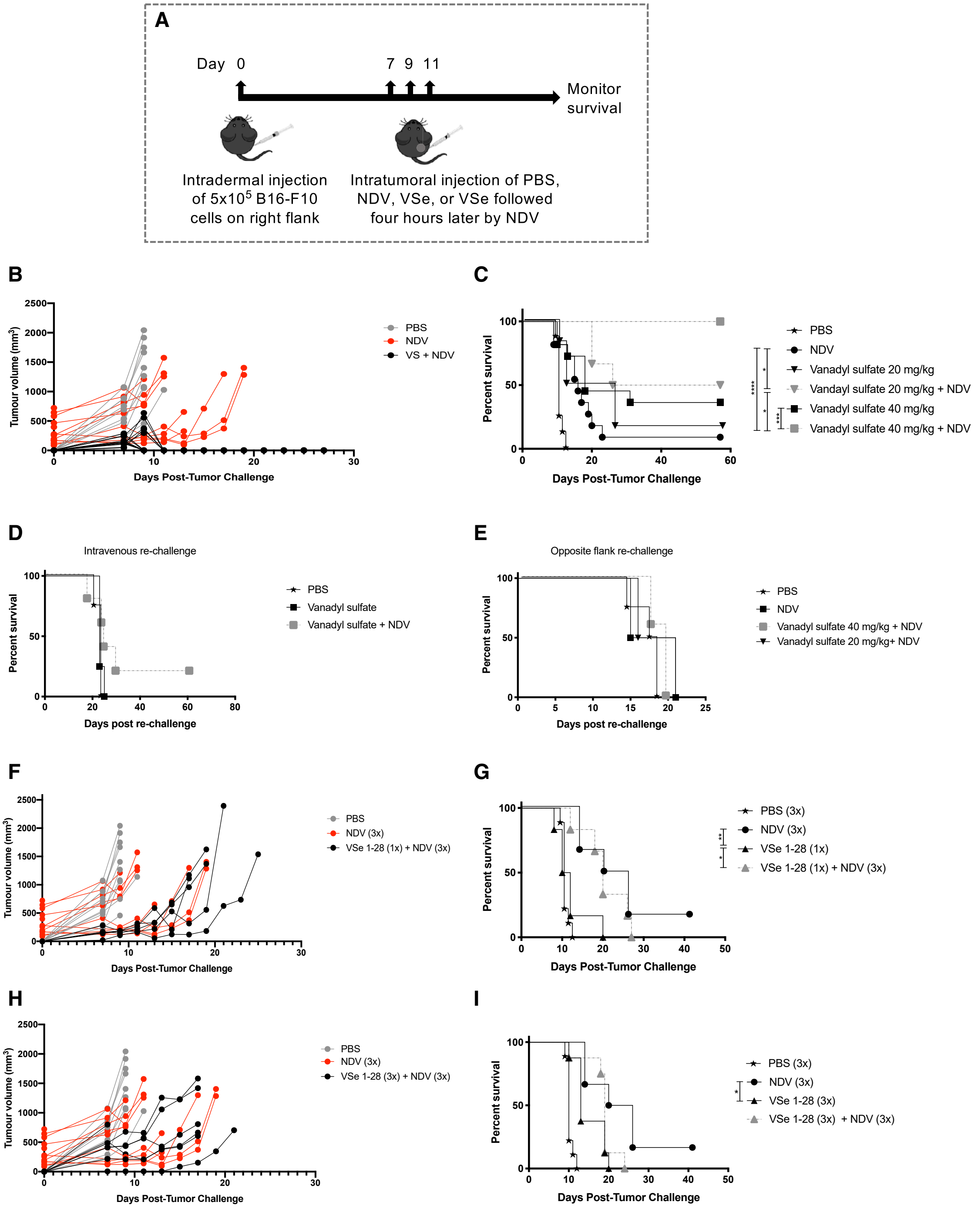

Figure 2. Effect of combination therapies on survival in the B16-F10 murine melanoma model

(A) Schematic of the experimental outline. B16-F10 cells (5×10^5 cells) were implanted intradermally. When tumors reached 5 mm in any direction, therapies were administered intratumorally (i.t.) every other day for a total of three treatments and tumor volume monitored. (B) Tumor volume of B16-F10 tumor-bearing mice treated three times with vanadyl sulfate (40 mg/kg) plus NDV, compared to PBS-treated tumor volumes. (C) C57BL/6 mice bearing B16-F10 intradermal tumors were treated intratumorally three times (48 h apart) with PBS, 5×10^7 PFU NDV, vanadyl sulfate (20 mg/kg or 40 mg/kg), or vanadyl sulfate 4 h prior to i.t. injection of 5×10^7 PFU NDV, and mice were monitored for survival. Significance was determined using the log rank (Mantel-Cox) test (*$p < 0.05$, **$p < 0.01$, ***$p < 0.001$, ****$p < 0.0001$). (D) Mice cured by treatment with vanadyl sulfate plus NDV were re-challenged with 5×10^5 B16-F10 cells intravenously to mimic a metastatic spread. No significant extension of survival was observed. (E) Survivors of the study in (C) were re-challenged with 5×10^5 B16-F10 cells on the opposite flank. No significant extension of survival was observed. (F and G) Tumor volume of

(legend continued on next page)

combination therapy stimulates B16-F10 cells to significantly increase the expression of IFN-β.

Survival of B16-F10 tumor-bearing mice is dramatically enhanced when vanadyl sulfate is administered in combination with NDV

Results of *in vitro* experiments prompted us to determine whether combining vanadyl sulfate plus NDV would translate into increased survival in an immunocompetent *in vivo* model. Six-week-old female C57BL/6 mice were implanted with 5×10^5 B16-F10 tumor cells intradermally. Tumors were measured using a digital caliper, and when they grew to 5 mm in diameter, treatment was initiated. Mice were injected intratumorally with phosphate-buffered saline (PBS), vanadyl sulfate (20 mg/kg or 40 mg/kg), NDV, or a combination of vanadyl sulfate + NDV every 48 h for a total of three treatments, with the combination group receiving vanadyl sulfate 4 h prior to injection with NDV (Figure 2A). Tumor volumes were measured every other day until they reached 15 mm in a single direction, which was defined as endpoint. We observed extremely rapid tumor regression in mice treated with vanadyl sulfate (40 mg/kg) plus NDV (Figure 2B) that was preceded by the formation of a thick, rigid scab that eventually resolved, typically within 5 days of the last treatment (Figure S2). Regressions of tumors in all mice that received the combination therapy were complete within only 96 h of the initiation of treatment. Remarkably, all mice in the vanadyl sulfate plus NDV treatment group were cured of their disease, as demonstrated by their failure to re-grow tumors by 60 days after the initial regression. When mice were treated with a lower dose of vanadyl sulfate (20 mg/kg) in combination with NDV, 50% of the mice were cured of their disease, showing the therapy had a dose-response effect (Figure 2C).

To evaluate whether this treatment induced a tumor-specific memory response, mice that were cured of their disease were re-challenged with 5×10^5 B16-F10 cells 60 days after the last PBS-treated mouse reached endpoint. Regardless of whether mice were re-challenged intravenously (to mimic metastatic disease) (Figure 2D) or subcutaneously on the opposite flank (Figure 2E), there was no significant increase in survival when compared to PBS-treated controls. These data demonstrate that although tumors treated with an optimal dose of vanadyl sulfate (i.e., 40 mg/kg) 4 h prior to injection with NDV regress rapidly and resolve completely, this specific combination treatment did not induce a sufficient memory response to control B16-F10 tumors, as mice were susceptible to re-challenge.

The pyrrole derivative, VSe 1-28, only modestly enhanced survival of B16-F10 tumor-bearing mice when used in combination with NDV

Given its published potential to enhance OV-mediated oncolysis,[35] we tested whether a single intratumoral dose of VSe 1-28 (40 mg/kg) or VSe 1-28 followed by three doses of 5×10^7 plaque-forming units (PFU) of intratumoral NDV every 48 h for a total of three treatments would enhance survival in B16-F10 tumor-bearing mice. Since VSe 1-28 has an enhanced half-life we initially investigated whether one dose of VSe 1-28 would be efficacious. As shown in Figure 2F, tumor growth was delayed but continually progressed. Similarly, in Figure 2G, there was a modest but significant increase in survival in the group receiving the combination treatment when compared to VSe 1-28 alone, but this did not prove to be curative. Given these results, we next tested whether administering three doses of the combination therapy 48 h apart, as was done with vanadyl sulfate, would yield better results. As shown in Figure 2H, tumor growth was impeded but, again, continually progressed. Results in Figure 2I show a similar trend in survival to the single-dose regimen. Therefore, there did not appear to be a benefit of increasing the number of doses of the combination therapy. Since VSe 1-28 did not potentiate NDV-mediated oncolytic virotherapy to nearly the extent that vanadyl sulfate did, we focused on the latter for subsequent mechanistic studies.

Vanadyl sulfate plus NDV combination therapy fails to increase the number of activated natural killer (NK) cells and tumor-specific T cells in circulation

To begin dissecting cellular mechanisms of action, we investigated the impact of the vanadyl sulfate NDV combination therapy on the activation status of both NK cells and T cells. Flow cytometric analysis of the number and activation status of NK cells in the blood was conducted 36 h after a single treatment of PBS, vanadyl sulfate (40 mg/kg), NDV (5×10^7 PFU/mL), or a combination of NDV plus vanadyl sulfate. In terms of the relative number of NK cells in circulation, mice treated with vanadyl sulfate alone had significantly higher numbers of $CD3^-$ $CD8^-$ $NK1.1^+$ cells in the blood compared to all other treatments (Figure 3A). We next quantified the relative number of activated $NK1.1^+$ cells based on expression of the early activation marker CD69 and found that the NDV-treated mice had significantly greater numbers of activated NK cells relative to mice treated with PBS or vanadyl sulfate alone and mice that received vanadyl sulfate plus NDV (Figure 3B). We observed similar results when we quantified the number of $NK1.1^+$ that were producing the effector cytokine IFN-γ (Figure 3C). These results suggest that while vanadyl sulfate treatment leads to an increase in the total number of NK cells in the blood, only mice that received NDV as a single agent had a significant increase in activated NK cells in the blood.

To evaluate tumor-specific T cell responses, the same groups of mice were bled 10 days after the first treatment and the amount of $CD8^+$ tumor-specific T cells in the blood was quantified using two different assays to identify B16-F10 tumor-specific T cells. The first assay is an antigen agnostic assay in which we exploit the fact that IFN-γ treatment leads to upregulation of major histocompatibility complex expression and thus display of putative

B16-F10 tumor-bearing mice treated once with PBS or once with VSe 1-28 (40 mg/kg) (F) and three times with 5×10^7 PFU NDV and monitored for survival (G). (H and I) Tumor volume of B16-F10 tumor-bearing mice treated three times with PBS or VSe 1-28 (40 mg/kg) and three times with 5×10^7 PFU NDV (H) and monitored for survival (I). Significance of survival was determined using log rank (Mantel-Cox) test (*p < 0.05, **p < 0.01, ***p < 0.001, ****p < 0.0001).

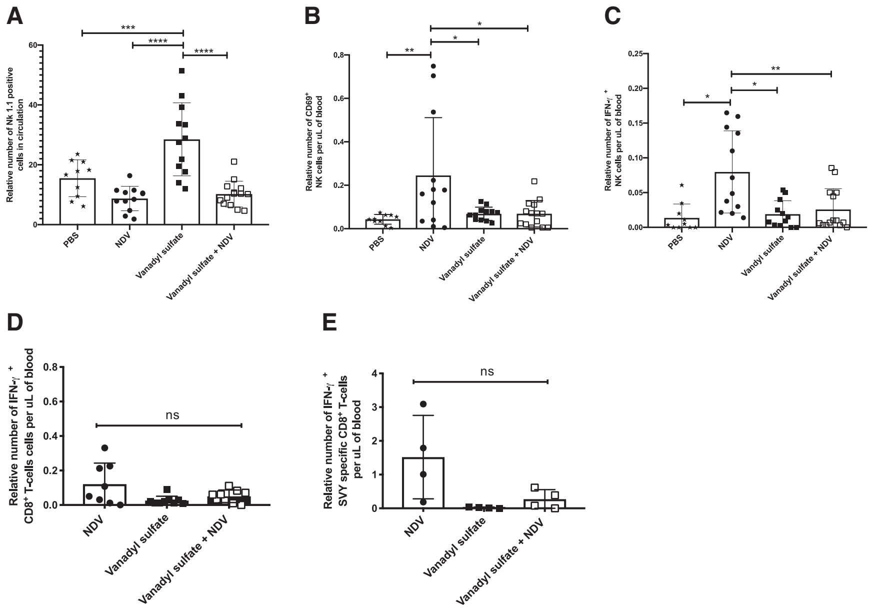

Figure 3. Characterization of immune cell populations in circulation 36 h post-treatment

(A–C) B16-F10 tumor-bearing mice were retro-orbitally bled 36 h after a single intratumoral treatment with PBS, NDV (5×10^7 PFU), vanadyl sulfate (40 mg/kg), or a combination of vanadyl sulfate plus NDV. Blood was processed and analyzed via fluorescence activated cell sorting (FACS) using a natural killer (NK) cell antibody panel. (D and E) Blood was drawn retro-orbitally from B16-F10 tumor-bearing mice 10 days after the first of three treatments was initiated and was processed and analyzed via FACS using a panel of antibodies to quantify CD8+ T cells responding to undefined tumor-associated antigens (D) and a tumor-specific antigen (SVY) (E). Significance was determined using a one-way ANOVA test (*$p < 0.05$, **$p < 0.01$, ***$p < 0.001$, ****$p < 0.0001$).

B16-F10 tumor-associated antigens. Tumor-specific T cells in the blood are then detected by flow cytometry after co-culture, as described.[37] The second assay we employed identified CD8$^+$ T cells specific for the melanoma-associated dopachrome tautomerase$_{180-188}$ peptide that is expressed by B16-F10 cells and is the dominant epitope for cytotoxic T cells in C57BL/6 mice. Regardless of the assay, none of the treatments significantly increased the number of B16-F10-specific CD8$^+$ T cells in the blood (Figures 3D and 3E). These data suggested that the mechanism(s) by which this combination treatment mediated its therapeutic effect could be better understood by investigating the effects it has on the tumor microenvironment (TME) and/or tumor-draining lymph nodes (TdLNs). Further, the rapidity of tumor regression and an apparent lack of induction of systemic tumor-specific T cells prompted an emphasis on assessments of innate immunity.

Treatment with vanadyl sulfate plus NDV enhances the number of activated NK cells in the tumor and TdLNs

To characterize the impact of combination treatment on the innate immune response occurring in the TME, tumors were harvested from B16-F10 tumor-bearing mice 36 h after a single intratumoral treatment with PBS, vanadyl sulfate (40 mg/kg), NDV, or a combination of vanadyl sulfate plus NDV. Our initial strategy was to treat tumors when they reached 5 mm in diameter and harvest them 36 h later; however, we observed that the tumors had dramatically receded in size and, in some cases, were too small for analysis. As such, we had to allow tumors to reach 8 mm in any one direction before administering the therapy. Tumors, TdLNs, and the same lymph nodes on the opposite flank were harvested 36 h after a single treatment, and various leukocyte subsets were quantified by flow cytometry. As shown in Figures 4A and 4B, the relative number of IFN-γ-producing NK cells in the tumor and TdLNs was significantly greater in the vanadyl sulfate plus NDV combination group compared to all other treatments. Interestingly, NDV as a single agent generated significantly more CD69$^+$ NK cells in the TME compared to all the other treatments (Figure 4C), whereas vanadyl sulfate plus NDV treatment generated significantly more CD69$^+$ NK cells in the TdLNs (Figure 4D).

To further elucidate the impact vanadyl sulfate plus NDV combination therapy has on dendritic cells (DCs), we quantified migratory (CD103$^+$) and lymphoid-resident (CD8α$^+$) classical DCs (cDCs)[38] in TdLNs. Additionally, we quantified the CCR7$^+$-expressing cDCs, as CCR7 has been shown to control different migratory events, including the homing of NK cells, T cells, and cDCs to lymphoid compartments.[39] A significant increase in the number of CD103$^+$ (Figure 4G), CD103$^+$CCR7$^+$ (Figure 4H), and CD8α$^+$CD103$^+$ (Figure 4I) cDCs were detected in the TdLNs of the vanadyl sulfate plus NDV treatment group compared to all other treatments. Of note, the CD8α$^+$ CD103$^+$ cDC1 lineage of cDCs is superior in antigen cross-presentation, a process of presenting exogenous antigens on major histocompatibility complex (MHC) class I to activate CD8$^+$ T cells and are thus particularly important for producing CD8$^+$ T cells that can kill tumor cells.[40] Taken together, combination therapy with vanadyl sulfate plus NDV significantly increases the number of activated NK cells in the tumor and the TdLNs as well as the total number of cDC1 cells in the TdLNs. These results suggest that vanadyl sulfate plus NDV combination therapy potentiates the innate immune response, which is probably why we see such rapid tumor regression.

Vanadyl sulfate plus NDV combination therapy promotes increased NK cell activation and reduces the number of M2 macrophages in the TME at acute time points

To elucidate the mechanism of action of vanadyl sulfate plus NDV combination therapy, we conducted a tumor-infiltrating lymphocyte (TIL) analysis using an antibody panel geared toward assessing the cytotoxic potential of NK cells and the presence of M2 macrophages. B16-F10 tumor-bearing mice were administered a single intratumoral

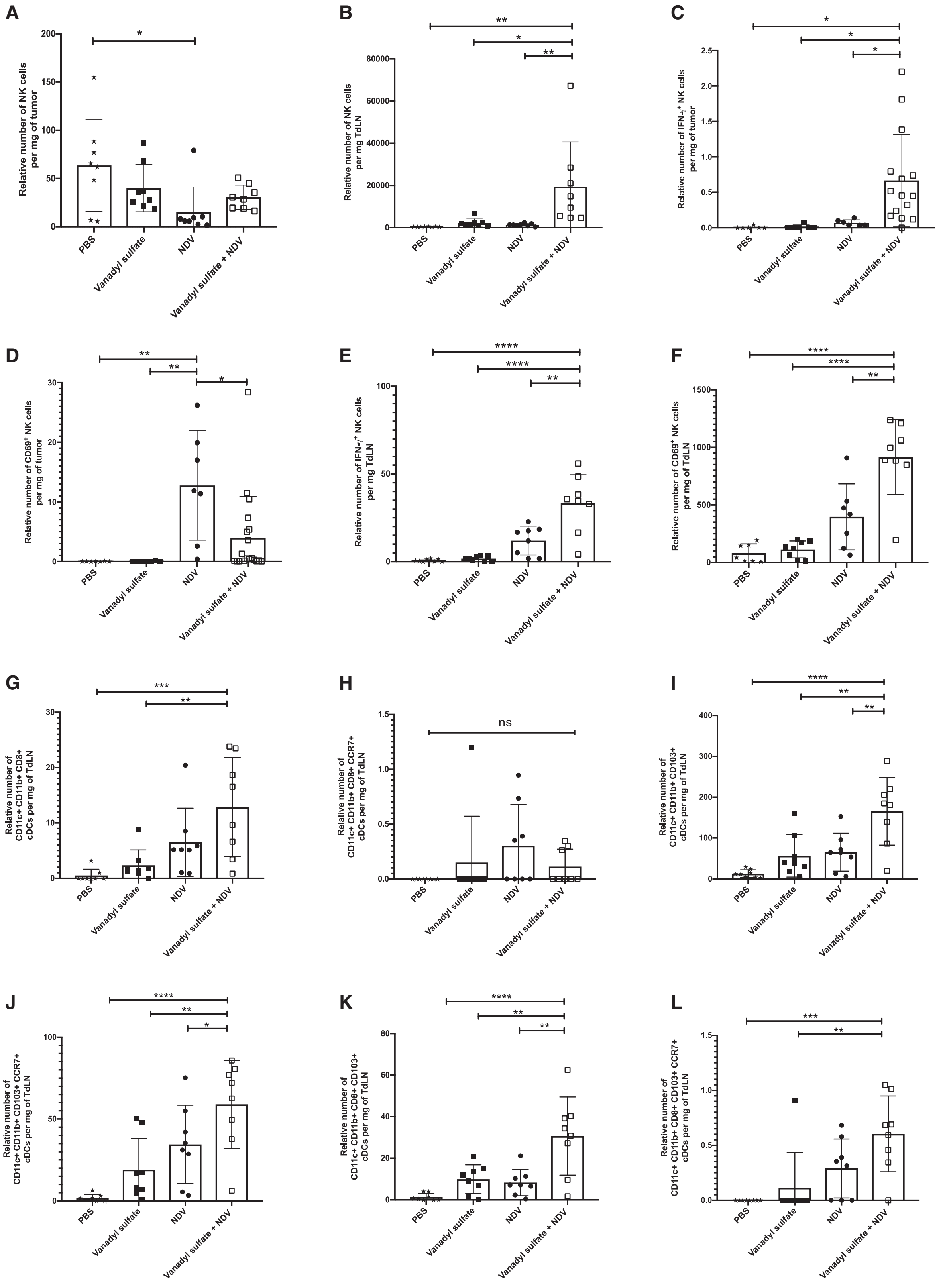

(legend on next page)

injection of PBS, vanadyl sulfate (40 mg/kg), NDV (5.0×10^7 PFU), or vanadyl sulfate 4 h prior to NDV. Tumors, which were 8×8 mm^2 at the initiation of treatment, were harvested at an acute time point (24 h), and immediately processed and stained with NK- and macrophage-specific antibody panels. The PBS-treated group contained the largest number of $NK1.1^+CD3^-$ cells compared to all other treatment groups (Figure 5A). However, it was observed that the highest percentage of NK cells producing granzyme B, a cytotoxic serine protease found in the granules of NK cells, was in the vanadyl sulfate plus NDV group, as it was significantly increased compared to PBS, vanadyl sulfate, and NDV treatment groups (Figures 5B and 5C). Moreover, the mean fluorescence intensity (MFI) of granzyme B, an indication of the density of expression of the molecules per cell, was also significantly increased in the vanadyl sulfate plus NDV treatment group compared to all other groups (Figure 5D). In addition, vanadyl sulfate plus NDV combination therapy significantly increased the percentage of IFN-γ-producing NK cells compared to all other groups, as shown in Figures 5E and 5F. Likewise, the MFI of IFN-γ was also significantly increased in NK cells from mice treated with vanadyl sulfate plus NDV compared to all other groups (Figure 5G).

We next examined the effect of vanadyl sulfate plus NDV combination therapy on tumor-associated macrophage populations, with macrophages defined as $CD11b^+F4/80^+$ cells. Specifically, we evaluated the number of M2 macrophages, identified through the presence of CD206, in B16-F10 tumor-bearing mice. As shown in Figures 5H and 5I, the percentage of $CD206^+$ macrophages was significantly reduced when vanadyl sulfate plus NDV therapy was administered compared to treatment with PBS or vanadyl sulfate. Alternatively, only vanadyl sulfate as a monotherapy had a significantly different MFI compared to macrophages in tumors derived from PBS-treated mice (Figure 5J). These data showcase the ability of vanadyl sulfate plus NDV combination therapy to enhance the activation and cytolytic potential of NK cells, while simultaneously decreasing the immunosuppressive nature of the TME through the reduction in the number of resident M2 macrophages.

Determination of cytokine profiles in tumors at an acute time point revealed significantly increased concentrations of IFN-β, GM-CSF, and monocyte chemoattractant protein-1 (MCP-1) in mice treated with vanadyl sulfate plus NDV

To better understand how this combination therapy might alter the cytokine profile in the TME at acute time points, thirteen different cytokines were quantified in B16-F10 tumors from mice treated once with PBS, vanadyl sulfate, or NDV, or a single dose of vanadyl sulfate plus NDV. Tumors were harvested 36 h after these treatments, and cytokines were quantified using a flow cytometry-based method. Vanadyl sulfate plus NDV combination therapy significantly increased IFN-β (Figure 6A), GM-CSF (Figure 6B), and MCP-1 (CCL2) (Figure 6C) protein concentrations compared to all other treatments. Treatment with NDV or vanadyl sulfate plus NDV both significantly increased CXCL-10 (Figure 6D) and CCL5 (Figure 6E) protein concentrations compared to tumors from mice treated with vanadyl sulfate or PBS alone. Finally, IL-6 (Figure 6F) was significantly increased and IL-1β (Figure 6G) was significantly decreased in the vanadyl sulfate plus NDV treatment group when compared to tumors from mice treated with PBS alone. Taken together, these results indicate that combining vanadyl sulfate with NDV leads to a rapid change in the cytokine profile in the TME, highlighted by a general increase in key immunomodulatory cytokines within the TME.

NK cells contributed to the therapeutic effect of vanadyl sulfate plus NDV combination therapy

Given the probable role of the innate immune system in mediating rapid tumor regression, and the apparent lack of a memory T cell response following vanadyl sulfate plus NDV treatment, NK and T cells were depleted in tumor-bearing mice according to the schedule in Figure 7A, and we monitored the effect this had on survival. In B16-F10 tumor-bearing mice depleted of NK cells using an anti-Asialo-GM1 antibody (Figure S3), the therapeutic efficacy of vanadyl sulfate plus NDV therapy was significantly reduced from 100% survival in the non-depleted mice to 12.5% survival in the mice depleted of NK cells (Figure 7B). Conversely, when B16-F10 tumor-bearing mice were depleted of T cells using anti-Thy1.2 (CD90.2) (Figure S4), there was a slight reduction in survival in the vanadyl sulfate plus NDV treatment group (Figure 7C), but this was not significant. Taken together, these depletion studies demonstrate that NK cells play a critical role in mediating the anti-tumor effect of vanadyl sulfate plus NDV combination therapy and suggest that T cells have a lesser impact on the de-bulking effect of this combination therapy.

Type I IFNs are fundamental in antitumor control and execute their function predominantly by modulating the activity of leukocytes. Notably, the impact of type I IFNs on NK cells is especially crucial for efficient tumor immunosurveillance.[41] Our data showed that vanadyl sulfate plus NDV combination therapy significantly increased IFN-β production in B16-F10 cells *in vitro* and *in vivo* in tumors (Figures 1F and 6B, respectively). To determine whether this increase in IFN-β production contributed to the enhanced antitumor efficacy of this combination therapy, we generated a recombinant NDV co-expressing the non-structural protein 1 (NS1) from the PR8 strain of influenza virus and GFP from two separate transcription units (NDV-NS1). NS1 has previously been shown to transiently suppress IFN-β production when expressed from a recombinant NDV.[42] As shown in Figure 7D, infection of cultured B16-F10 cells with NDV-NS1 significantly reduced IFN-β production relative to the parental NDV. When NDV-NS1 was administered intratumorally to B16-F10 tumor-bearing mice in combination with vanadyl sulfate there

Figure 4. Quantification of activated tumor-infiltrating lymphocytes

(A–L) Tumor (A, B, E, and F) and TdLNs (C, D, and G–L) were harvested from B16-F10 tumor-bearing mice 36 h after a single intratumoral treatment with PBS, vanadyl sulfate (40 mg/kg), NDV (5×10^7 PFU), or a combination of vanadyl sulfate plus NDV and then the quantity and quality of NK cells (A–D) and dendritic cells (E–L) characterized by FACs. Significance was determined using a one-way ANOVA test (*p < 0.05, **p < 0.01, ***p < 0.001, ****p < 0.0001).

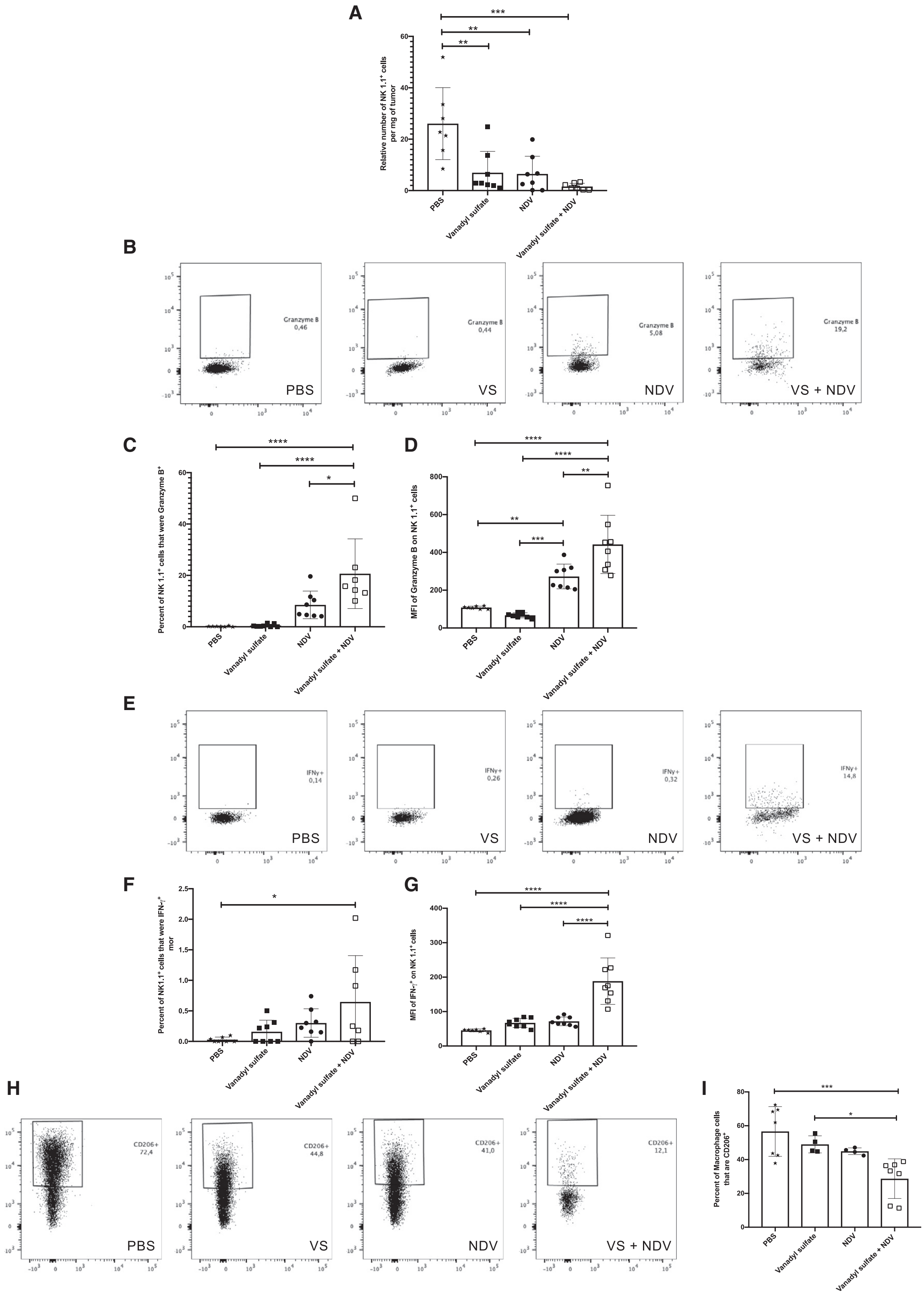

(legend on next page)

was a significant reduction in efficacy, as only 37.5% of mice survived compared to 100% of mice in the vanadyl sulfate plus NDV treatment group (Figure 7E). Of note, there was no significant difference in survival between NDV and NDV-NS1 when the viruses were administered as monotherapies (Figure 7D). These data highlight the importance of IFN-β in mediating the rapid tumor regression observed in the vanadyl sulfate plus NDV combination therapy.

Efficacy of vanadyl sulfate plus NDV combination therapy was not limited to a single cancer type

Since intratumoral injection of B16-F10 melanomas with vanadyl sulfate plus NDV resulted in rapid tumor regression and long-term cures, we wanted to investigate whether these results could be recapitulated in a cancer type other than melanoma. Murine RM9[43] prostate cancer cells (1×10^5) were injected subcutaneously into the left flank of C57BL/6 male mice. Approximately 7 days later, when tumors reached ~5 mm in diameter, PBS, vanadyl sulfate (40 mg/kg), NDV (5×10^7 PFU), or a combination of vanadyl sulfate and NDV was administered intratumorally and tumor growth monitored. As shown in Figure 8, treatment with vanadyl sulfate plus NDV significantly enhanced the survival of RM9 tumor-bearing mice compared to all other treatment groups. These findings demonstrate that this combination therapy has the ability to enhance survival in other cancer models.

DISCUSSION

In this study, we show that intratumoral administration of the pan-tyrosine phosphatase inhibitor, vanadyl sulfate, 4 h prior to injection with oncolytic NDV significantly potentiates the innate antitumor immune response leading to rapid tumor regression, with curative potential for orthotopic melanoma. The efficacy of this combination therapy appears to be dependent on IFN-β production and is mediated in large part by NK cells.

There are numerous reports in the literature highlighting the ability of OVs to convert an immunosuppressive "cold" TME to a "hotter" immunostimulatory one; however, thus far, OVs have had limited activity as single agents in clinical trials.[44] To date, an assortment of OV-drug combination therapies have been shown to act in additive or synergistic fashion, resulting in increased therapeutic effects not possible with either therapy alone.[45,46] Here we sought to determine whether two small-molecule VSes, vanadyl sulfate and VSe 1-28, which were previously shown to significantly improve the therapeutic efficacy of VSVΔ51 in a range of tumor models,[21,35] would potentiate NDV-mediated oncolysis in a murine melanoma model. While both VSes significantly reduced the metabolic activity of B16-F10 melanoma cells *in vitro* when combined with NDV, neither combination led to substantially increased virus replication or transgene expression. It is possible that the combination of vanadyl sulfate plus NDV reduced the viability of B16-F10 cells by rendering them more permissive to NDV-induced apoptosis or cell death. Since vanadyl sulfate has been shown to activate several transcription factors, including NF-κB,[47] as well as influence the activity of the cell cycle, oncogenes, and tumor suppressor genes,[32] it is conceivable that altered intracellular signaling could have potentiated the cytotoxic effects of oncolytic NDV.[48] Additionally, vanadium compounds have been shown to induce single-stranded DNA breaks leading to genotoxic stress preferentially in cancer cells.[49] Finally, vanadate has been shown to enhance NDV-mediated cell fusion,[50] which could potentially increase the direct oncolytic effect of NDV and induce immunogenic cell death.[51]

The ability of vanadyl sulfate plus NDV combination therapy to increase the production of IFN-β, particularly in the TME, is intriguing. Despite its role in innate antiviral immune responses, IFN-β has been shown to support activation of antitumor immunity when expressed in VSV,[52] vaccinia virus,[53] and measles virus.[54] It is also well known that IFN-β plays a critical role in increasing the cytotoxic activity of NK cells, as impaired IFN signaling in this subset results in a substantial reduction in mature NK cells expressing receptors such as CD11b, CD122, and Ly49, leading to impaired cytotoxic activity.[41,55]

An intratumoral dose of 40 mg/kg vanadyl sulfate was chosen based on the fact that it was previously shown to significantly enhance tumor regression when used in combination with oncolytic VSVΔ51.[21] While there was no evidence of toxicity when 40 mg/kg vanadyl sulfate was delivered intratumorally or intraperitoneally, IV administration of either 40 mg/kg or 20 mg/kg vanadyl sulfate was acutely toxic to mice.

It is important to point out that vanadyl sulfate alone was able to induce significant tumor regression and enhance survival of B16-F10 tumor-bearing mice, with ~50% of mice being cured of their tumors. The mechanism behind this warrants further investigation, as well as dose escalation studies to determine whether greater survival rates are achievable. However, while vanadyl sulfate as a monotherapy significantly enhanced survival, it pales in comparison to the 100% survival observed when vanadyl sulfate was combined with NDV.

When B16-F10 tumors were treated with a combination of vanadyl sulfate plus NDV, tumor regression occurred rapidly, within only 48–96 h after the last treatment. The rapid nature with which tumors

Figure 5. Quantification of cytotoxic NK cells and M2 macrophages in the B16-F10 tumor micro-environment (TME) at an acute time point

Tumors were harvested from B16-F10 tumor-bearing mice 24 h after a single intratumoral treatment with PBS, vanadyl sulfate (40 mg/kg), NDV (5.0×10^7 PFU), or a combination of vanadyl sulfate plus NDV. (A) Relative number of NK 1.1^+ $CD3^-$ cells. (B) Dot plot representatives of each treatment group for granzyme B^+ NK 1.1^+ $CD3^-$ cells. (C) Percent of NK 1.1^+ $CD3^-$ cells that are granzyme B^+ in the TME. (D) Mean fluorescence intensity (MFI) of granzyme B per cell in NK 1.1^+ $CD3^-$ cells in the TME. (E) Representative dot plots for each treatment group for $IFN\gamma^+$ NK 1.1^+ $CD3^-$. (F) Percentage of NK 1.1^+ $CD3^-$ cells that are $IFN\gamma^+$ in the TME. (G) MFI of $IFN\gamma^+$ per cell in NK 1.1^+ $CD3^-$ cells. (H) Representative dot plots for each treatment group for F4/80^+ $CD11b^+$ $CD206^+$ cells. (I) Percentage of F4/80^+ $CD11b^+$ $CD206^+$ cells in the TME. Significance was determined using a one-way ANOVA test (*$p < 0.05$, **$p < 0.01$, ***$p < 0.001$, ****$p < 0.0001$).

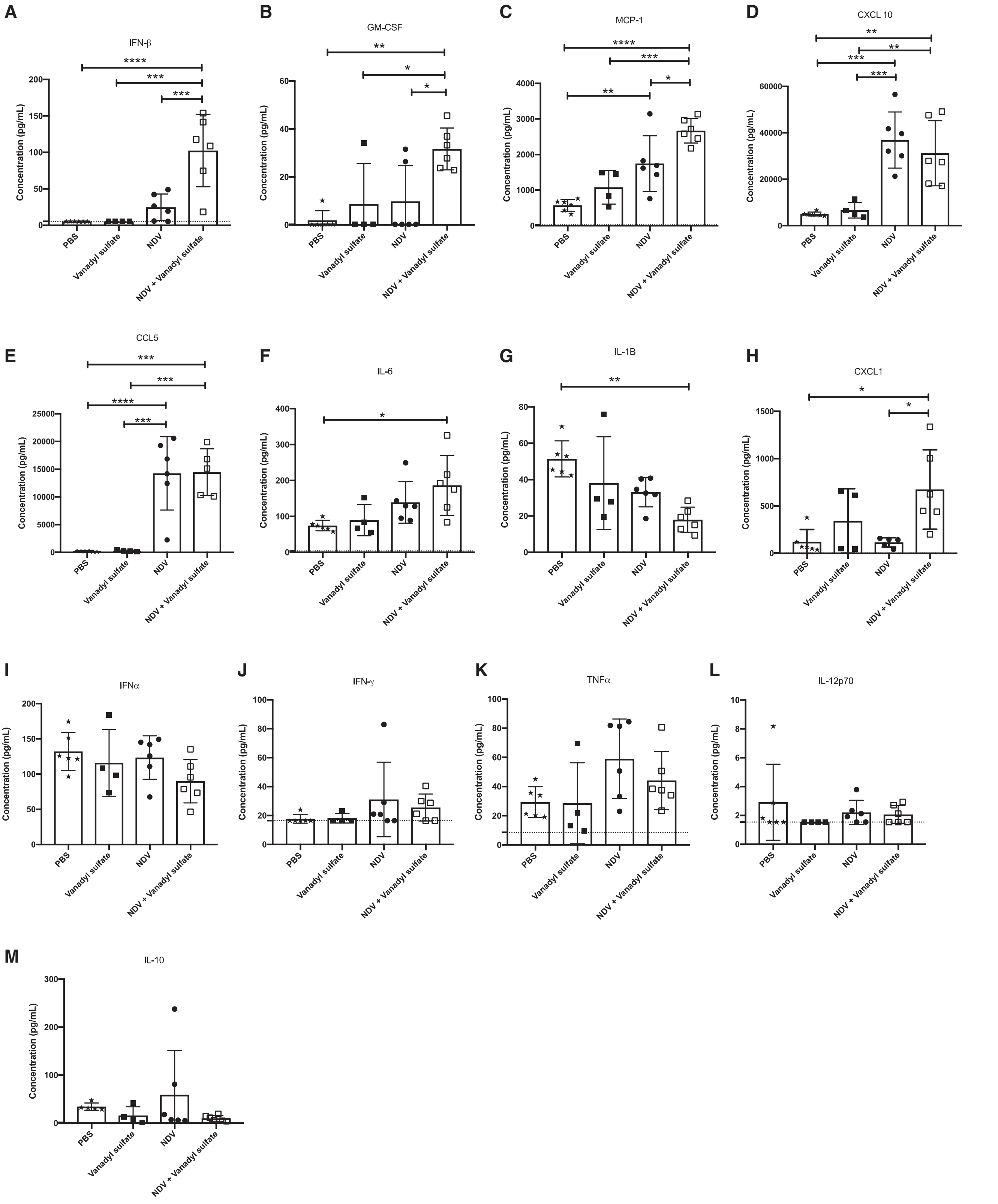

(legend on next page)

regressed suggested that the therapeutic effect of this combination therapy is mediated in large part by the innate immune system, as an effective adaptive immune response was unlikely to have been generated within this time frame.[56] This interpretation is further supported by the fact that mice that were cured of their tumors after receiving the combination vanadyl sulfate plus NDV therapy succumbed to re-challenge, suggesting they did not generate a sufficient anti-tumor memory response. It is possible that failure to induce adaptive tumor-specific T cell responses was due to tumors regressing so rapidly that there was insufficient stimulation of naive T cells, but this remains to be determined.

In contrast to vanadyl sulfate, when the VSe 1-28[35] was administered either as a single agent or in combination with NDV, the increase in survival was of much lower magnitude in the orthotopic melanoma model. This was consistent whether tumor-bearing mice received one or three doses of the combination therapy. Considering that VSe 1-28 has been shown to transcriptionally repress genes associated with a type I IFN response,[35] it could be speculated that these results support the notion that the magnitude of type I IFN signaling correlates with the therapeutic efficacy of combination therapies involving a VSe and NDV.

To better understand the role the innate immune system plays upon administration of vanadyl sulfate and NDV, we evaluated the ability of this combination therapy to modulate NK cells in circulation after a single administration. Although vanadyl sulfate significantly increased the number of $CD3^{-}$ $CD8^{-}$ $NK1.1^{+}$ cells in circulation, if NDV was included in the treatment regimen there was a dramatic reduction in the number of NK cells in the blood, likely due to the well-recognized phenomenon of virus-induced lymphopenia.[57–59] It is plausible that intratumoral administration of NDV led to increased NK cell trafficking to the TME, and, as such, their numbers in circulation were reduced. Indeed, when B16-F10 TILs were analyzed 36 h after a single treatment, the number of IFNγ-producing NK cells was significantly increased in the combination therapy group compared to all other groups. It is important to note that for the TIL analysis it was necessary to allow tumors to reach 8 mm in any one dimension before initiating treatment, as smaller tumors (e.g., ~5 × 5 mm) treated with vanadyl sulfate plus NDV were dramatically reduced in size by 36 h, making it impossible to harvest a sufficient number of cells to conduct TIL analysis. In addition to the tumor, the TdLNs also had significantly increased numbers of IFNγ-producing NK cells in the vanadyl sulfate plus NDV treatment group, suggesting that activated NK cells might be an important mediator of the antitumor efficacy of this therapy. Conversely, the number of $CD69^{+}$ NK cells in the tumor 36 h post-treatment was only significantly increased when NDV was administered as a monotherapy; however, in the TdLNs there was a significant increase in the number of $CD69^{+}$ NK cells in the combination therapy treatment group, which could be due to increased NK cell trafficking to the TdLNs. An alternative explanation for this difference may be because NK cells express the CD69 activation marker after encountering stimuli such as IL-12p70 or IFN-α, neither of which was upregulated in the TME of the combination therapy treatment group at the 36-h time point.[60] Further investigation into the impact of vanadyl sulfate plus NDV combination therapy on the cytotoxic potential of NK cells revealed the highest percentage of granzyme B^{+} NK cells in the tumor in the combination therapy group at the 24 h time point. A similar trend was observed for $IFN\gamma^{+}$ NK cells; however, the fact that there were more $IFN\gamma^{+}$ NK cells in the TdLNs of the combination therapy group at 36 h post-treatment suggests the possibility that activated NK cells are trafficking to the TdLNs after exerting their cytolytic activity. Myeloid-derived suppressor cells (MDSCs) are innate leukocytes that secrete cytokines such as transforming growth factor beta (TGF-β), IL-10, and IL-6, which can suppress NK cell activity.[61] Vanadyl sulfate plus NDV therapy reduced the number of tumor-associated macrophages (TAMs), specifically M2 macrophages, which may have led to a reduction in immunosuppression and enhanced NK cell activity.

In the TME of B16-F10 tumors treated with a combination of vanadyl sulfate and NDV, MCP-1 (CCL2), which is a chemoattractant for monocytes and macrophages, GM-CSF, which stimulates proliferation of granulocyte and macrophage progenitor cells and enhances the activity of conventional type 1 DCs,[62] a unique subset of DCs that have been shown to enhance local cytotoxic T cell functions,[63] were significantly increased relative to all other treatment groups. Additionally, IFN-β, which has anti-proliferative and pro-apoptotic effects on tumor cells,[64–66] was significantly increased relative to all other treatment groups. As a testament to their importance in strengthening the anti-tumor immune response, a variety of OVs, including HSV,[67,68] vaccinia virus,[69] and adenovirus,[70] have been engineered to express these exact pro-inflammatory cytokines, resulting in enhanced anti-tumor immunity. While genetic modifications that support both adaptive (e.g., GM-CSF) and innate (e.g., IFN-β) anti-tumor immunity have been applied to improve OV therapy,[61] it may be possible to achieve a similar effect without having to engineer NDV by combining it with vanadyl sulfate at the time of administration. The combination of vanadyl sulfate plus NDV also resulted in a significant decrease in IL-1β, a cytokine that has been shown to play a key role in carcinogenesis and tumor progression in the B16-F10 hepatic metastasis model.[71] Finally, combining vanadyl sulfate with NDV did not diminish NDV's natural ability to increase expression of chemokines CXCL10 (IP-10) and CCL5 (RANTES), which are important for the chemoattraction of $CD8^{+}$ T lymphocytes.[72,73] Taken together, the cytokine signature induced by the vanadyl sulfate plus NDV combination therapy would be expected to increase chemotaxis of effector leukocytes and, through the action of IFN-β, enhance NK cell-mediated anti-tumor cytotoxicity.

Figure 6. Quantification of cytokine and chemokine expression levels in B16-F10 tumors 36 h post-treatment

(A–M) B16-F10 tumor-bearing mice were administered a single intratumoral injection of PBS, vanadyl sulfate (40 mg/kg), NDV (5.0×10^{7} PFU), or a combination of vanadyl sulfate and NDV. Thirty-six hours after treatment, tumors were harvested, homogenized, and then analyzed using the LEGENDplex Mouse Anti-Virus Response assay. Significance was determined using a one-way ANOVA test (*p < 0.05, **p < 0.01, ***p < 0.001, ****p < 0.0001).

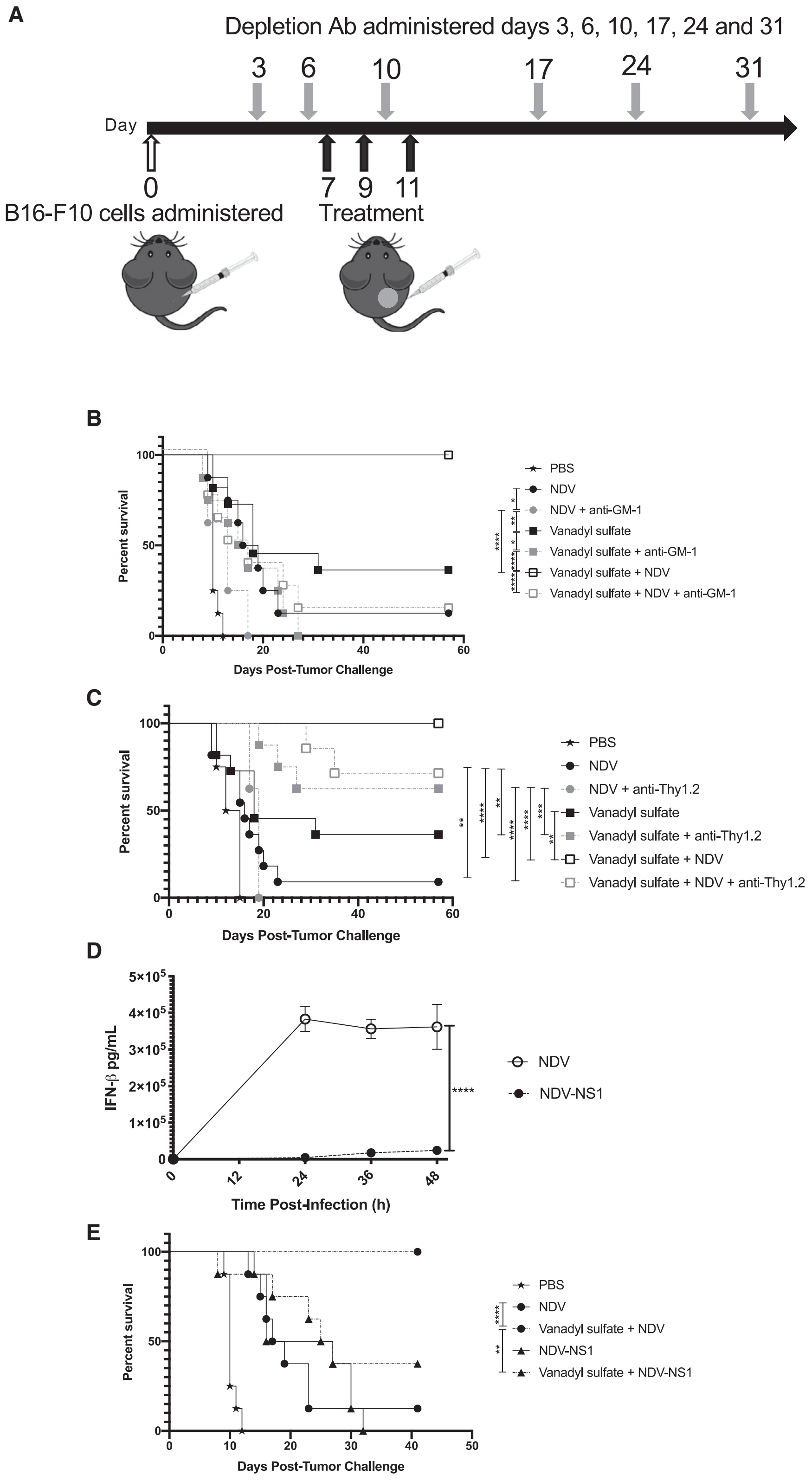

Figure 7. Impact of immune cell depletion on survival

(A) Experimental outline showing the timing of NK cell depletion antibody treatments, T cell depletion antibody treatments (gray arrows), and therapeutic treatments (PBS, vanadyl sulfate [40 mg/kg], NDV [5×10^7 PFU], or vanadyl sulfate plus NDV; black arrows) in mice implanted with 5×10^5 B16-F10 cells intradermally. (B) Survival of B16-F10 tumor-bearing NK cell-depleted mice treated i.t. with PBS, vanadyl sulfate, NDV, or vanadyl sulfate plus NDV. (C) Survival of B16-F10 tumor-bearing T cell-depleted mice treated intratumorally with PBS, vanadyl sulfate, NDV, or vanadyl sulfate plus NDV. (D) IFN-β quantification of B16-F10 cells treated with NDV or NDV-NS1 (MOI = 1), made relative to cell-only control. Significance was determined using a 2-way ANOVA test (*$p < 0.05$, **$p < 0.01$, ***$p < 0.001$, ****$p < 0.0001$). (E) Survival of B16-F10 tumor-bearing mice treated intratumorally with PBS, vanadyl sulfate, NDV, NDV-NS1, or a combination of vanadyl sulfate plus NDV or NDV-NS1 (5×10^7 PFU). Significance was determined using log rank (Mantel-cox) test (*$p < 0.05$, **$p < 0.01$, ***$p < 0.001$, ****$p < 0.0001$).

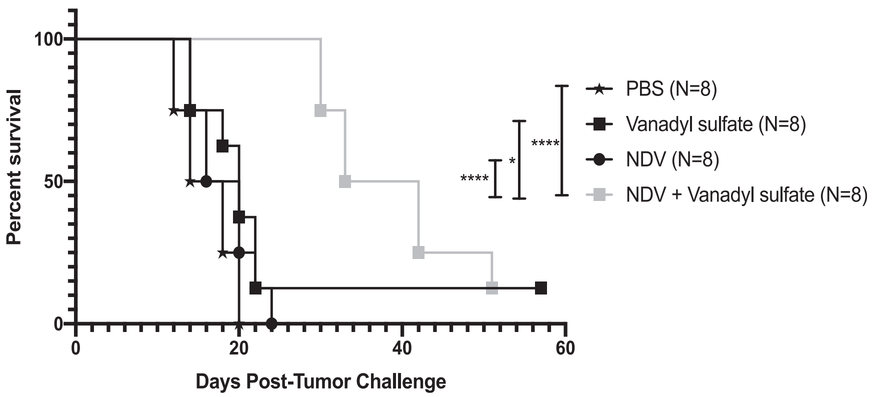

Figure 8. Impact of vanadyl sulfate plus NDV combination therapy in a subcutaneous RM9 mouse model

Male C57BL/6 mice were administered syngeneic 1 × 10^5 RM9 prostate cancer cells subcutaneously. Seven days later, mice received phosphate-buffered saline (i.t.), vanadyl sulfate (40 mg/kg; i.t.), NDV (5.0 × 10^7 PFU; i.t.) or vanadyl sulfate intraperitoneally (i.p.) 4 h prior to i.t. administration of NDV. Mice were euthanized when tumors reached 15 mm in any one direction. Significance was determined using log rank (Mantel-Cox) test (*p < 0.05, **p < 0.01, ***p < 0.001, ****p < 0.0001).

When NK cells were depleted, there was a marked decrease in efficacy in the vanadyl sulfate plus NDV combination therapy group. While disease progression increased in the absence of NK cells in all therapeutic settings, this is likely because both vanadyl sulfate and NDV enhance NK cell activity when used as monotherapies, but when used in a combination they have an additive or synergistic effect to enhance the antitumor effects mediated by NK cells. Conversely, when T cells were depleted, there was comparatively less impact on efficacy in the combination treatment group. The observation of complete cures following this regimen in a large proportion of mice in the absence of T cells highlights the role of the innate immune system in mediating tumor regression. Immunosuppressive regulatory T cells (Tregs) that are present in the TME have been shown to express the Thy1.2 marker; as such, the use α-Thy1.2 may have resulted in a reduction of Tregs, resulting in a more immunogenic TME.[74] Furthermore, hepatic $Thy1^+$ NK cells have been shown to play an important role in viral clearance, antibacterial immunity, and cytotoxicity toward tumor cells, albeit this is a low-density marker.[75–77] As such, α-Thy1.2 may inhibit activated NK cells bearing the Thy1 marker, and the reduction in survival of the vanadyl sulfate plus NDV treatment group when α-Thy1.2 was used to deplete T cells may be attributed to a reduction in NK cell numbers. Regardless, the NK cell depletion study clearly suggests that this subset was a dominant effector in this model. Previous studies using oncolytic VSV in the CT26 model in combination with vanadyl sulfate attributed enhanced antitumor effects to increased T cell infiltration and activation, wherein efficacy was abrogated in T cell-deficient mice.[21] Taken together, the sum total of these findings suggests that the dominant cellular mechanism of action leading to robust efficacy of OV/vanadyl sulfate combination treatments may differ depending on the specific OV and/or tumor model. Alternatively, it might be explained by the kinetics of tumor regression; if the regression is rapid enough, there may be no need for the immune system to mount an adaptive response.

The effects of VSes on replication of NDV and induction of type I IFN appear to be somewhat opposite from what was previously observed with VSV (i.e., VSe 1-28 increased VSV replication,[20,35] while vanadium compounds decreased type I IFN[21]). This highlights that combination partners may lead to unexpected results within the context of different viruses, even within the viruses in the same genetic taxon (i.e., *Mononegavirales*). NDV is an avian paramyxovirus that is very different from the rhabdovirus VSV. NDV replicates poorly in mammalian cells,[78] particularly murine cells,[14] whereas VSV is highly replicative in a wide range of cell lines.[79] The mechanism of action of oncolytic NDV is primarily through immune stimulation[80] rather than extensive virus replication and destruction of tumor cells, which may contribute to the differences observed.

Type I IFNs are important regulators of innate leukocytes such as NK cells and DCs in anti-cancer host responses.[41] Type I IFNs can directly enhance NK cell-mediated cytotoxicity and cytokine production.[81,82] However, the effector functions of NK cells need to be "licensed," which is predominantly carried out by type I IFN through the stimulation of DCs to produce IL-15, which is a crucial cytokine for NK cell development, proliferation, and function.[83–86] Given that combination treatment with vanadyl sulfate plus NDV significantly increased IFN-β production both *in vitro* and *in vivo*, we wanted to further dissect the role of type I IFN in the antitumor function of this combination therapy. Widespread and constitutive inhibition of interferon-α/β receptor (IFNAR) signaling, as in the case of $IFNAR^{-/-}$ mice or the use of IFNAR-blocking antibodies, would be expected to have pleiotropic effects on tumor growth kinetics and leukocyte function. Therefore, we engineered a recombinant NDV to express the influenza PR8 NS1 protein so as to localize depletion of type I IFN within the TME.[51] When NDV-NS1 was combined with vanadyl sulfate in the B16-F10 survival model, a significant loss of efficacy was observed (i.e., a reduction in long-term survivors from 100% to 37.5%), but not to the same extent as NK cell depletion (i.e., a reduction in long-term survivors to 12.5%). The reduction in IFN-β expression may have resulted in decreased activation of NK cells, resulting in a lower efficacy; however, when NK cells are depleted, efficacy is severely impacted. Thus, the sustained antitumor response of this combination therapy is dependent upon the presence of both intact type I IFN signaling and NK cell functions.

Importantly, treatment with vanadyl sulfate plus NDV extended survival beyond that achieved with monotherapies in the context of

subcutaneous prostate tumors, demonstrating the potential for this combination therapy to treat cancers other than melanoma.

A variety of therapeutic strategies, including prime-boost vaccination[87] and infected cell vaccines,[88] have extended survival in the B16-F10 model primarily through the induction of an anti-tumor adaptive immune response mediated in large part by cytotoxic $CD8^+$ T cells. In contrast, the efficacy of vanadyl sulfate plus NDV combination therapy is mediated in large part by the innate arm of the immune system, mainly NK cells. An intriguing future direction of this research might be to combine vanadyl sulfate with a cytotoxic T cell-centric oncolytic viroimmunotherapy in an attempt to harness the full potential of the immune system.

Although combining NDV with vanadyl sulfate was able to generate durable cures, there are some limitations with this approach that identify other possible future directions. The combination of vanadyl sulfate plus NDV led to the complete regression of tumors typically within 4 days of treatment, which may be too short of a time frame for the development of an effective anti-tumor adaptive immune response. The incorporation of a transgene that stimulates an adaptive immune response, such as IL-2, may increase the ability of this combination therapy to induce a memory $CD8^+$ T cell response.[89] In addition, marrying this therapy with another strategy to stimulate the adaptive immune response, for example immune checkpoint blockade, might remedy this pitfall. Additionally, optimizing a method by which vanadyl sulfate could be administered intravenously could greatly broaden the applicability of this therapy, by allowing for systemic administration and potentially increasing the amount of vanadyl sulfate that enters the tumors.[90] One possibility is to conjugate vanadyl sulfate to nanoparticles designed for optimal delivery to tumors to safely deliver the VSe to the TME.[91,92] The successful delivery of vanadyl sulfate, at a relevant dose, by intravenous administration would enhance the clinical translation of this therapy and increase the value of this technology. Lastly, we used a highly fusogenic strain of NDV in this study; however, demonstration that vanadyl sulfate is efficacious when combined with lentogenic NDV or inactivated NDV would prove valuable, as mesogenic NDV is a select agent in the United States. As such, the potential additive or synergistic effects of vanadyl sulfate and lentogenic NDV/inactivated NDV warrants further investigation.

In summary, we identified a novel combination therapy that involves administering vanadyl sulfate in conjunction with NDV, leading to rapid and sustained tumor regression in mice bearing orthotopic B16-F10 tumors. This therapeutic strategy generates a robust innate immune response in which NK cells and type I IFN signaling are dominant effector mechanisms. This treatment not only increases the number of activated NK cells in the TME and TdLNs but also upregulates cytokines associated with activation and trafficking of monocytes, neutrophils, and macrophages and suppression of TAMs. Moreover, this combination therapy is efficacious in the absence of T cells. These findings may prove useful in potentiating the innate immune response to induce rapid regression of primary melanomas and warrants further investigation in other pre-clinical models of cancer, and in the context of non-fusogenic strains of NDV or strains of NDV engineered to enhance a T cell memory response, to facilitate translation of this therapeutic strategy into the clinic. Alternatively, the potency of this therapy in the absence of T cells suggests it could be of utility in the treatment of cancers in patients with compromised adaptive immune systems.

MATERIALS AND METHODS

Ethics approval

All mouse experiments were performed in compliance with the guidelines set forth by the Canadian Council on Animal Care. The Animal Care Committee at the University of Guelph approved all methods. Randomly allocated groups of 6-week-old female C57BL/6 mice (for the B16-F10 model) and 6-week-old male C57BL/6 mice (for the RM9 model) purchased from Charles River Laboratories (Wilmington, MA, USA) were housed at the University of Guelph in a specific pathogen-free animal isolation facility. Mice were housed in groups of four and food (Teklad Global 14% Protein Rodent Maintenance Diet, Indianapolis, IN, USA) and tap water were provided *ad libitum*. Mice were acclimated to the environment for 7 days prior to study initiation.

Cell culture

DF-1 chicken embryo fibroblast cells (ATCC CRL-12203), B16-F10 murine melanoma cells (ATCC CRL-6475), and RM9 murine prostate cancer cells (ATCC CRL-3312) were purchased from ATCC. Cells were maintained in Dulbecco's modified Eagle's medium, supplemented with 10% bovine calf serum and 2 mM L-glutamine, and grown in a humidified incubator at 37°C in 5% CO_2-95% air. All cell lines were continually tested for mycoplasma using a MycoAlert mycoplasma detection kit (Lonza cat no. LT07-118; Morrisville, NC, USA).

NDV production

The NDVF3aa-GFP genome was engineered to include an amino acid substitution in the fusion protein, of the 289th amino acid from leucine to alanine. Second, NDV was made to express the full-length enhanced GFP protein, with the GFP gene located between the phosphoprotein (P) and matrix (M) genes of NDV. The helper plasmids (pTM1-NP, pTM1-P, and pTM1-L) were a kind gift from Dr. Peter Palese (Mount Sinai, NY, USA). Plasmids were purified with the GenElute HP Plasmid Maxiprep Kit (Sigma-Aldrich). Recombinant NDV-GFP was rescued and propagated in specific pathogen-free eggs (Canadian Food and Inspection Agency), and allantoic fluid was harvested 50 h post-inoculation and clarified by centrifugation (1,500 $\times$ g for 10 min at 4°C) and purified as described.[93] The virus was aliquoted and stored at −80°C and subsequently referred to as "NDV."

NDV-NS1

The mouse codon-optimized NS1 gene from the A/Puerto Rico/8/1934 strain of influenza A virus (GenBank: 956533) was synthesized as a gene block by GenScript. The gene block was cloned into the NDVF3aa (L289A) GFP cDNA between the GFP and M gene using

InFusion cloning techniques. Each transgene was flanked by a gene start, intergenic, and gene stop sequence, as well as preceded by a Kozak sequence. Therefore, NDV expresses GFP and NS1 from two different transcriptional cassettes to generate NDV-NS1, which was then rescued using the strategy described above. Note that NS1 had no impact on virus titers. NDV-NS1 titers in eggs were comparable if not greater than NDV-GFP when titered on DF1 cells.

Evaluating the effect of VSes on NDV production

B16-F10 cells were plated at a density of 1×10^4 cells per well in 96-well tissue culture plates. At 24 h post-seeding, a range of concentrations of VSe 1-28 (a kind gift from Dr. J.S. Diallo) or vanadyl sulfate (Sigma-Aldrich, cat no. 233706) were administered to cells, and 4 h later cells were infected with NDV at an MOI of 0.01. At 48 h post-infection, supernatants were collected and clarified, and 10-fold serial dilutions were administered to DF-1 cells seeded in 96-well plates. Fluorescence of GFP was evaluated using a Carl Zeiss Axio 154 Observer A1 inverted fluorescence microscope 96 h later, and the titer of the virus was determined using a $TCID_{50}$ assay.

$TCID_{50}$ assay

Virus titer was determined by $TCID_{50}$ assay and expressed as $TCID_{50}$/mL. Briefly, NDV samples were serially diluted 10-fold with Dulbecco's PBS and inoculated onto DF-1 cells seeded at 70%–80% confluency in 96-well cell culture plates. After 96 h, green fluorescence was observed using a Carl Zeiss Axio 154 Observer A1 inverted fluorescence microscope, and titer was calculated according to the Spearman-Kärber method.[94]

Resazurin assay

Resazurin (7-hydroxy-3*H*-phenoxazin-3-one 10-oxide) was purchased from Sigma-Aldrich (USA) and dissolved in PBS (pH 7.0). Cell viability was evaluated by treating cells with 10 μM of resazurin and incubating for 4–6 h at 37°C, 5% CO_2. Excitation was measured at a wavelength of 535 nm and emission at a wavelength of 590 nm, using a BioTek Synergy HT plate reader (Winooski, VT, USA).

GFP quantification

B16-F10 cells were seeded on a 96-well tissue culture plate at 5×10^4 cells per well. GFP expression was quantified 48 h post-infection using a PerkinElmer Multimode plate reader. Fluorescence intensity was quantified at an excitation wavelength of 500 nm and emission wavelength of 600 nm.

Quantification of murine IFN-β

B16-F10 cells seeded in 12-well tissue culture plates were treated with a VSe for 4 h followed by infection with NDV at a MOI of 0.01. When determining the impact of NS1 expression on IFN-β production, an MOI of 1 was used. Cell supernatants were collected at varying time points post-infection and IFN-β production quantified using the LumiKine Xpress ELISA (InvivoGen), as per the manufacturer's instructions. Concentrations of IFN-β were normalized to untreated controls.

Survival experiments

Six-week-old female C57BL/6 mice were injected with 5×10^5 B16-F10 cells in a final volume of 20 μL (diluted in PBS) intra-dermally. In the case of the RM9 model, 6-week-old male C57BL/6 mice were injected with 1×10^5 RM9 cells in a 30 μL volume subcutaneously. When tumors reached 5 × 5 mm, vehicle control or a VSe was injected intratumorally in a 20 μL volume. In the combination therapy groups, 5×10^7 PFU of NDV was administered intratumorally 4 h later. Tumor volumes were measured every other day using an electronic caliper. Ear clipping patterns allowed for accurate tracking of individual mice. Tumor volume was calculated using this formula: volume = (length × width2)/2. Mice were euthanized when tumors reached 15 mm in any direction.

NK cell analyses

The following antibodies were used for flow cytometric analysis: Brilliant Violet (BV) 421 anti-mouse CD3 (BioLegend, cat no. 100336, clone 145-2C11), fluorescein isothiocyanate (FITC) anti-mouse CD69 (BD Bioscience, cat no. 553236, clone H1.2F3), allophycocyanin (APC) anti-mouse NK1.1 (BD Bioscience, cat no. 550627), Phycoerythrin (PE) anti-mouse IFNγ (BioLegend, cat no. 505807, clone XMG1.2), and Fc Block-CD16/32 (BioLegend, cat no. 101320, clone 93). A second TIL utilized FITC anti-mouse granzyme B (BioLegend, cat no. 515403, clone GB11), Alexa Fluor 488 anti-mouse CD11b (BioLegend cat no. 101219, clone M1/70), APC anti-mouse F4/80 (BioLegend, cat no123115, cloneBM8), and FITC anti-mouse CD206 (BioLegend cat no. 141703, clone C068C2) antibodies. Cells were isolated from blood, tumors, and TdLNs and processed for surface labeling with antibodies against CD3, NK1.1, and CD69, in a single staining panel, followed by the application of fixable viability dye Zombie NIR (BioLegend) to label dead cells and intracellular staining with an antibody against IFNγ after fixation with intracellular fixation buffer (eBioscience, cat no. 00-8222-49). Data were acquired using a FACS Canto II flow cytometer with FACSDiva version 8.0 software (BD Biosciences) and analyzed using FlowJo version 10.6.2 software (BD Biosciences). Cell counts were normalized to volume of blood drawn or to tumors by using the weight (mg) of tumor after its excision from mice. See Figures S5–S9 for gating strategies.

Tumor-specific T cell analysis

B16-F10 cells seeded at a density of 1×10^5 cells per well in a round-bottom 96-well plate was stimulated with 50 units of murine IFN-γ recombinant protein (eBioscience, cat no. 14-8311-63, San Diego, CA, USA) for 48 h. Blood was drawn 10 days after the first dose of PBS, vanadyl sulfate (40 mg/kg), VSe 1-28 (40 mg/kg), NDV (5.0×10^7 PFU/mL), or a combination of VSe and NDV and applied to IFN-γ-stimulated B16-F10 cells as previously described.[46] Samples were then split into two groups, and one group was pulsed with the $DCT^{180-188}$ peptide, which is the immunodominant B16-F10 tumor-derived epitope for C57BL/6 mice. Cells were then treated with Fc Block-CD16/32 (BioLegend, cat no. 101320, clone 93), stained and analyzed by flow cytometry after surface staining with antibodies against CD3, FITC anti-mouse CD4 (eBioscience, cat no. 11-0043-85, clone RM4-4), and BV 510 anti-mouse CD8 (BioLegend, cat

no. 100752, clone 53-6.7), and intracellular staining against IFN-γ. Cell counts were normalized to volume of blood drawn or to tumors by using the weight (mg) of tumor after its excision from mice.

Quantification of cytokines and chemokines

Proteins were isolated from tumors or TdLNs of B16-F10 tumor-bearing C57BL/6 mice 36 h after their first treatment of PBS, vanadyl sulfate, NDV, or vanadyl sulfate plus NDV. Radioimmunoprecipitation (RIPA) buffer (25 mM Tris-HCl [pH 7.6], 150 mM NaCl, 1% NP-40, 1% sodium deoxycholate, 0.1% SDS) and tissue homogenizer beads (MP Biomedicals, cat no. 6910-100) were added to sample tubes. A Precellys 24 tissue homogenizer (Bertin Instruments) was used to homogenize tissue at 6,000 rpm for two intervals of 60 s. Samples were then centrifuged at 14,000 rpm (Thermo Scientific Micro-Click 30 × 2 Fixed Angle Microtube Rotor) for 15 min at 4°C and the supernatant collected. Samples were then analyzed with the LEGENDplex Mouse Antiviral Response kit (cat no. 740261; BioLegend,) according to the manufacturer's protocol.

Depletion of leukocytes

Six-week-old female C57BL/6 mice were administered 5×10^5 B16-F10 cells in a 20 uL volume intradermally and at days 3, 6, 10, 17, 24, and 31 post-challenge, Ultra-LEAF purified anti-Asialo-GM1 (Poly21460; BioLegend) for NK cell depletion, or anti-mouse Thy1.2 (CD90.2; clone 30H12; Bio X Cell) for T cell depletion, was administered into the intraperitoneal cavity in a 250 μL volume. Therapies were typically administered on days 7, 9, and 11 after tumor cell implantation.

SUPPLEMENTAL INFORMATION

Supplemental Information can be found online at https://doi.org/10.1016/j.omto.2021.01.009.

ACKNOWLEDGMENTS

We thank all those who participated in the care of the animals at the University of Guelph Animal Isolation Unit. This work was funded by the Natural Sciences and Engineering Research Council of Canada through a Discovery Grant (grant no. 355661) to S.K.W. and an operating grant that was jointly funded by the Cancer Research Society (grant no. 23534) and Canadian Institutes of Health Research-Institute of Cancer Research (grant no. 159395) to B.W.B. and S.K.W. This work was supported in part through the Canadian Scientific Research and Experimental Development program. T.M.M. was recipient of an OVC PhD Scholarship.

AUTHOR CONTRIBUTIONS

T.M.M., J.P.v.V., R.A., L.A.S., P.M.M., L.S., K.K., B.W.B., J.S.D., and S.K.W. contributed to the design of the project. T.M.M., J.P.v.V., L.A.S., M.M.G., A.D.R., E.M.F., and J.C.I. performed the experiments. T.M.M. and S.K.W. drafted the manuscript, and all authors read, edited, and approved the manuscript.

DECLARATION OF INTERESTS

J.S.D. is an inventor on a patent describing the use of vanadium compounds as enhancers of oncolytic virotherapy. This patent (publication #20190231832) is licensed to Virica Biotech, where J.S.D. is co-founder and serves as an executive.

REFERENCES

1. Kaufman, H.L., Kohlhapp, F.J., and Zloza, A. (2015). Oncolytic viruses: a new class of immunotherapy drugs. Nat. Rev. Drug Discov. *14*, 642–662.
2. Zheng, M., Huang, J., Tong, A., and Yang, H. (2019). Oncolytic Viruses for Cancer Therapy: Barriers and Recent Advances. Mol. Ther. Oncolytics *15*, 234–247.
3. Elankumaran, S., Rockemann, D., and Samal, S.K. (2006). Newcastle disease virus exerts oncolysis by both intrinsic and extrinsic caspase-dependent pathways of cell death. J. Virol. *80*, 7522–7534.
4. Mansour, M., Palese, P., and Zamarin, D. (2011). Oncolytic specificity of Newcastle disease virus is mediated by selectivity for apoptosis-resistant cells. J. Virol. *85*, 6015–6023.
5. Krishnamurthy, S., Takimoto, T., Scroggs, R.A., and Portner, A. (2006). Differentially regulated interferon response determines the outcome of Newcastle disease virus infection in normal and tumor cell lines. J. Virol. *80*, 5145–5155.
6. Fiola, C., Peeters, B., Fournier, P., Arnold, A., Bucur, M., and Schirrmacher, V. (2006). Tumor selective replication of Newcastle disease virus: association with defects of tumor cells in antiviral defence. Int. J. Cancer *119*, 328–338.
7. Hines, N.L., and Miller, C.L. (2012). Avian paramyxovirus serotype-1: a review of disease distribution, clinical symptoms, and laboratory diagnostics. Vet. Med. Int. *2012*, 708216.
8. Burman, B., Pesci, G., and Zamarin, D. (2020). Newcastle Disease Virus at the Forefront of Cancer Immunotherapy. Cancers (Basel) *12*, 3552.
9. Vigil, A., Park, M.S., Martinez, O., Chua, M.A., Xiao, S., Cros, J.F., Martínez-Sobrido, L., Woo, S.L., and García-Sastre, A. (2007). Use of reverse genetics to enhance the oncolytic properties of Newcastle disease virus. Cancer Res. *67*, 8285–8292.
10. Altomonte, J., Marozin, S., Schmid, R.M., and Ebert, O. (2010). Engineered newcastle disease virus as an improved oncolytic agent against hepatocellular carcinoma. Mol. Ther. *18*, 275–284.
11. Janke, M., Peeters, B., de Leeuw, O., Moorman, R., Arnold, A., Fournier, P., and Schirrmacher, V. (2007). Recombinant Newcastle disease virus (NDV) with inserted gene coding for GM-CSF as a new vector for cancer immunogene therapy. Gene Ther. *14*, 1639–1649.
12. Xu, X., Sun, Q., Yu, X., and Zhao, L. (2017). Rescue of nonlytic Newcastle Disease Virus (NDV) expressing IL-15 for cancer immunotherapy. Virus Res. *233*, 35–41.
13. Pühler, F., Willuda, J., Puhlmann, J., Mumberg, D., Römer-Oberdörfer, A., and Beier, R. (2008). Generation of a recombinant oncolytic Newcastle disease virus and expression of a full IgG antibody from two transgenes. Gene Ther. *15*, 371–383.
14. Zamarin, D., Holmgaard, R.B., Ricca, J., Plitt, T., Palese, P., Sharma, P., Merghoub, T., Wolchok, J.D., and Allison, J.P. (2017). Intratumoral modulation of the inducible co-stimulator ICOS by recombinant oncolytic virus promotes systemic anti-tumour immunity. Nat. Commun. *8*, 14340.
15. Vijayakumar, G., Palese, P., and Goff, P.H. (2019). Oncolytic Newcastle disease virus expressing a checkpoint inhibitor as a radioenhancing agent for murine melanoma. EBioMedicine *49*, 96–105.
16. Vijayakumar, G., McCroskery, S., and Palese, P. (2020). Engineering Newcastle Disease Virus as an Oncolytic Vector for Intratumoral Delivery of Immune Checkpoint Inhibitors and Immunocytokines. J. Virol. *94*, e01677-19.
17. Zamarin, D., Holmgaard, R.B., Subudhi, S.K., Park, J.S., Mansour, M., Palese, P., Merghoub, T., Wolchok, J.D., and Allison, J.P. (2014). Localized oncolytic virotherapy overcomes systemic tumor resistance to immune checkpoint blockade immunotherapy. Sci. Transl. Med. *6*, 226ra32.
18. Zamarin, D., and Wolchok, J.D. (2014). Potentiation of immunomodulatory antibody therapy with oncolytic viruses for treatment of cancer. Mol. Ther. Oncolytics *1*, 14004.
19. Fessas, P., Possamai, L.A., Clark, J., Daniels, E., Gudd, C., Mullish, B.H., Alexander, J.L., and Pinato, D.J. (2020). Immunotoxicity from checkpoint inhibitor therapy: clinical features and underlying mechanisms. Immunology *159*, 167–177.

20. Diallo, J.S., Le Boeuf, F., Lai, F., Cox, J., Vaha-Koskela, M., Abdelbary, H., MacTavish, H., Waite, K., Falls, T., Wang, J., et al. (2010). A high-throughput pharmacoviral approach identifies novel oncolytic virus sensitizers. Mol. Ther. *18*, 1123–1129.

21. Selman, M., Rousso, C., Bergeron, A., Son, H.H., Krishnan, R., El-Sayes, N.A., Varette, O., Chen, A., Le Boeuf, F., Tzelepis, F., et al. (2018). Multi-modal Potentiation of Oncolytic Virotherapy by Vanadium Compounds. Mol. Ther. *26*, 56–69.

22. Selman, M., Ou, P., Rousso, C., Bergeron, A., Krishnan, R., Pikor, L., Chen, A., Keller, B.A., Ilkow, C., Bell, J.C., and Diallo, J.S. (2018). Dimethyl fumarate potentiates oncolytic virotherapy through NF-κB inhibition. Sci. Transl. Med. *10*, eaao1613.

23. Arulanandam, R., Batenchuk, C., Varette, O., Zakaria, C., Garcia, V., Forbes, N.E., Davis, C., Krishnan, R., Karmacharya, R., Cox, J., et al. (2015). Microtubule disruption synergizes with oncolytic virotherapy by inhibiting interferon translation and potentiating bystander killing. Nat. Commun. *6*, 6410.

24. Jennings, V.A., Scott, G.B., Rose, A.M.S., Scott, K.J., Migneco, G., Keller, B., Reilly, K., Donnelly, O., Peach, H., Dewar, D., et al. (2019). Potentiating Oncolytic Virus-Induced Immune-Mediated Tumor Cell Killing Using Histone Deacetylase Inhibition. Mol. Ther. *27*, 1139–1152.

25. Berghauser Pont, L.M., Balvers, R.K., Kloezeman, J.J., Nowicki, M.O., van den Bossche, W., Kremer, A., Wakimoto, H., van den Hoogen, B.G., Leenstra, S., Dirven, C.M., et al. (2015). In vitro screening of clinical drugs identifies sensitizers of oncolytic viral therapy in glioblastoma stem-like cells. Gene Ther. *22*, 947–959.

26. Nguyên, T.L., Abdelbary, H., Arguello, M., Breitbach, C., Leveille, S., Diallo, J.S., Yasmeen, A., Bismar, T.A., Kirn, D., Falls, T., et al. (2008). Chemical targeting of the innate antiviral response by histone deacetylase inhibitors renders refractory cancers sensitive to viral oncolysis. Proc. Natl. Acad. Sci. USA *105*, 14981–14986.

27. Crans, D.C., Mahroof-Tahir, M., and Keramidas, A.D. (1995). Vanadium chemistry and biochemistry of relevance for use of vanadium compounds as antidiabetic agents. Mol. Cell. Biochem. *153*, 17–24.

28. Bevan, A.P., Drake, P.G., Yale, J.F., Shaver, A., and Posner, B.I. (1995). Peroxovanadium compounds: biological actions and mechanism of insulin-mimesis. Mol. Cell. Biochem. *153*, 49–58.

29. Heffetz, D., Bushkin, I., Dror, R., and Zick, Y. (1990). The insulinomimetic agents H2O2 and vanadate stimulate protein tyrosine phosphorylation in intact cells. J. Biol. Chem. *265*, 2896–2902.

30. Cohen, N., Halberstam, M., Shlimovich, P., Chang, C.J., Shamoon, H., and Rossetti, L. (1995). Oral vanadyl sulfate improves hepatic and peripheral insulin sensitivity in patients with non-insulin-dependent diabetes mellitus. J. Clin. Invest. *95*, 2501–2509.

31. Pierce, L.M., Alessandrini, F., Godleski, J.J., and Paulauskis, J.D. (1996). Vanadium-induced chemokine mRNA expression and pulmonary inflammation. Toxicol. Appl. Pharmacol. *138*, 1–11.

32. Tsave, O., Petanidis, S., Kioseoglou, E., Yavropoulou, M.P., Yovos, J.G., Anestakis, D., Tsepa, A., and Salifoglou, A. (2016). Role of Vanadium in Cellular and Molecular Immunology: Association with Immune-Related Inflammation and Pharmacotoxicology Mechanisms. Oxid. Med. Cell. Longev. *2016*, 4013639.

33. Pisano, M., Arru, C., Serra, M., Galleri, G., Sanna, D., Garribba, E., Palmieri, G., and Rozzo, C. (2019). Antiproliferative activity of vanadium compounds: effects on the major malignant melanoma molecular pathways. Metallomics *11*, 1687–1699.

34. Guerrero-Palomo, G., Rendón-Huerta, E.P., Montaño, L.F., and Fortoul, T.I. (2019). Vanadium compounds and cellular death mechanisms in the A549 cell line: The relevance of the compound valence. J. Appl. Toxicol. *39*, 540–552.

35. Dornan, M.H., Krishnan, R., Macklin, A.M., Selman, M., El Sayes, N., Son, H.H., Davis, C., Chen, A., Keillor, K., Le, P.J., et al. (2016). First-in-class small molecule potentiators of cancer virotherapy. Sci. Rep. *6*, 26786.

36. Czekanska, E.M. (2011). Assessment of cell proliferation with resazurin-based fluorescent dye. Methods Mol. Biol. *740*, 27–32.

37. van Vloten, J.P., Santry, L.A., McAusland, T.M., Karimi, K., McFadden, G., Petrik, J.J., Wootton, S.K., and Bridle, B.W. (2019). Quantifying Antigen-Specific T Cell Responses When Using Antigen-Agnostic Immunotherapies. Mol. Ther. Methods Clin. Dev. *13*, 154–166.

38. Jiao, Z., Bedoui, S., Brady, J.L., Walter, A., Chopin, M., Carrington, E.M., Sutherland, R.M., Nutt, S.L., Zhang, Y., Ko, H.J., et al. (2014). The closely related CD103+ dendritic cells (DCs) and lymphoid-resident CD8+ DCs differ in their inflammatory functions. PLoS ONE *9*, e91126.

39. Pesce, S., Moretta, L., Moretta, A., and Marcenaro, E. (2016). Human NK Cell Subsets Redistribution in Pathological Conditions: A Role for CCR7 Receptor. Front. Immunol. *7*, 414.

40. Merad, M., Sathe, P., Helft, J., Miller, J., and Mortha, A. (2013). The dendritic cell lineage: ontogeny and function of dendritic cells and their subsets in the steady state and the inflamed setting. Annu. Rev. Immunol. *31*, 563–604.

41. Müller, L., Aigner, P., and Stoiber, D. (2017). Type I Interferons and Natural Killer Cell Regulation in Cancer. Front. Immunol. *8*, 304.

42. Zamarin, D., Martínez-Sobrido, L., Kelly, K., Mansour, M., Sheng, G., Vigil, A., García-Sastre, A., Palese, P., and Fong, Y. (2009). Enhancement of oncolytic properties of recombinant newcastle disease virus through antagonism of cellular innate immune responses. Mol. Ther. *17*, 697–706.

43. Thompson, T.C., Southgate, J., Kitchener, G., and Land, H. (1989). Multistage carcinogenesis induced by ras and myc oncogenes in a reconstituted organ. Cell *56*, 917–930.

44. Chiu, M., Armstrong, E.J.L., Jennings, V., Foo, S., Crespo-Rodriguez, E., Bozhanova, G., Patin, E.C., McLaughlin, M., Mansfield, D., Baker, G., et al. (2020). Combination therapy with oncolytic viruses and immune checkpoint inhibitors. Expert Opin. Biol. Ther. *20*, 635–652.

45. Wennier, S.T., Liu, J., and McFadden, G. (2012). Bugs and drugs: oncolytic virotherapy in combination with chemotherapy. Curr. Pharm. Biotechnol. *13*, 1817–1833.

46. Ribas, A., Dummer, R., Puzanov, I., VanderWalde, A., Andtbacka, R.H.I., Michielin, O., Olszanski, A.J., Malvehy, J., Cebon, J., Fernandez, E., et al. (2017). Oncolytic Virotherapy Promotes Intratumoral T Cell Infiltration and Improves Anti-PD-1 Immunotherapy. Cell *170*, 1109–1119.e10.

47. Imbert, V., Rupec, R.A., Livolsi, A., Pahl, H.L., Traenckner, E.B., Mueller-Dieckmann, C., Farahifar, D., Rossi, B., Auberger, P., Baeuerle, P.A., and Peyron, J.F. (1996). Tyrosine phosphorylation of I kappa B-alpha activates NF-kappa B without proteolytic degradation of I kappa B-alpha. Cell *86*, 787–798.

48. Wozniak, K., and Blasiak, J. (2004). Vanadyl sulfate can differentially damage DNA in human lymphocytes and HeLa cells. Arch. Toxicol. *78*, 7–15.

49. Rojas, E., Valverde, M., Herrera, L.A., Altamirano-Lozano, M., and Ostrosky-Wegman, P. (1996). Genotoxicity of vanadium pentoxide evaluate by the single cell gel electrophoresis assay in human lymphocytes. Mutat. Res. *359*, 77–84.

50. Yamamoto, F., Fujioka, H., Iinuma, M., Takano, M., Maeno, K., Nagai, Y., and Ito, Y. (1984). Enhancement of Newcastle disease virus-induced fusion of mouse L cells by sodium vanadate. Microbiol. Immunol. *28*, 75–83.

51. Krabbe, T., and Altomonte, J. (2018). Fusogenic Viruses in Oncolytic Immunotherapy. Cancers (Basel) *10*, 216.

52. Willmon, C.L., Saloura, V., Fridlender, Z.G., Wongthida, P., Diaz, R.M., Thompson, J., Kottke, T., Federspiel, M., Barber, G., Albelda, S.M., and Vile, R.G. (2009). Expression of IFN-beta enhances both efficacy and safety of oncolytic vesicular stomatitis virus for therapy of mesothelioma. Cancer Res. *69*, 7713–7720.

53. Kirn, D.H., Wang, Y., Le Boeuf, F., Bell, J., and Thorne, S.H. (2007). Targeting of interferon-beta to produce a specific, multi-mechanistic oncolytic vaccinia virus. PLoS Med. *4*, e353.

54. Li, H., Peng, K.W., Dingli, D., Kratzke, R.A., and Russell, S.J. (2010). Oncolytic measles viruses encoding interferon beta and the thyroidal sodium iodide symporter gene for mesothelioma virotherapy. Cancer Gene Ther. *17*, 550–558.

55. Swann, J.B., Hayakawa, Y., Zerafa, N., Sheehan, K.C., Scott, B., Schreiber, R.D., Hertzog, P., and Smyth, M.J. (2007). Type I IFN contributes to NK cell homeostasis, activation, and antitumor function. J. Immunol. *178*, 7540–7549.

56. Miao, H., Hollenbaugh, J.A., Zand, M.S., Holden-Wiltse, J., Mosmann, T.R., Perelson, A.S., Wu, H., and Topham, D.J. (2010). Quantifying the early immune response and adaptive immune response kinetics in mice infected with influenza A virus. J. Virol. *84*, 6687–6698.

57. Schobesberger, M., Summerfield, A., Doherr, M.G., Zurbriggen, A., and Griot, C. (2005). Canine distemper virus-induced depletion of uninfected lymphocytes is associated with apoptosis. Vet. Immunol. Immunopathol. *104*, 33–44.

58. Salonen, R., Ilonen, J., and Salmi, A.A. (1989). Measles virus inhibits lymphocyte proliferation in vitro by two different mechanisms. Clin. Exp. Immunol. *75*, 376–380.

59. O'Donnell, D.R., and Carrington, D. (2002). Peripheral blood lymphopenia and neutrophilia in children with severe respiratory syncytial virus disease. Pediatr. Pulmonol. *34*, 128–130.

60. Borrego, F., Robertson, M.J., Ritz, J., Peña, J., and Solana, R. (1999). CD69 is a stimulatory receptor for natural killer cell and its cytotoxic effect is blocked by CD94 inhibitory receptor. Immunology *97*, 159–165.

61. Melcher, A., Parato, K., Rooney, C.M., and Bell, J.C. (2011). Thunder and lightning: immunotherapy and oncolytic viruses collide. Mol. Ther. *19*, 1008–1016.

62. Arellano, M., and Lonial, S. (2008). Clinical uses of GM-CSF, a critical appraisal and update. Biologics *2*, 13–27.

63. Cancel, J.C., Crozat, K., Dalod, M., and Mattiuz, R. (2019). Are Conventional Type 1 Dendritic Cells Critical for Protective Antitumor Immunity and How? Front. Immunol. *10*, 9.

64. Chawla-Sarkar, M., Leaman, D.W., and Borden, E.C. (2001). Preferential induction of apoptosis by interferon (IFN)-beta compared with IFN-alpha2: correlation with TRAIL/Apo2L induction in melanoma cell lines. Clin. Cancer Res. *7*, 1821–1831.

65. Johns, T.G., Mackay, I.R., Callister, K.A., Hertzog, P.J., Devenish, R.J., and Linnane, A.W. (1992). Antiproliferative potencies of interferons on melanoma cell lines and xenografts: higher efficacy of interferon beta. J. Natl. Cancer Inst. *84*, 1185–1190.

66. Wong, V.L., Rieman, D.J., Aronson, L., Dalton, B.J., Greig, R., and Anzano, M.A. (1989). Growth-inhibitory activity of interferon-beta against human colorectal carcinoma cell lines. Int. J. Cancer *43*, 526–530.

67. Parker, J.N., Meleth, S., Hughes, K.B., Gillespie, G.Y., Whitley, R.J., and Markert, J.M. (2005). Enhanced inhibition of syngeneic murine tumors by combinatorial therapy with genetically engineered HSV-1 expressing CCL2 and IL-12. Cancer Gene Ther. *12*, 359–368.

68. Franke, V., Berger, D.M.S., Klop, W.M.C., van der Hiel, B., van de Wiel, B.A., Ter Meulen, S., Wouters, M.W.J.M., van Houdt, W.J., and van Akkooi, A.C.J. (2019). High response rates for T-VEC in early metastatic melanoma (stage IIIB/C-IVM1a). Int. J. Cancer *145*, 974–978.

69. Kim, J.H., Oh, J.Y., Park, B.H., Lee, D.E., Kim, J.S., Park, H.E., Roh, M.S., Je, J.E., Yoon, J.H., Thorne, S.H., et al. (2006). Systemic armed oncolytic and immunologic therapy for cancer with JX-594, a targeted poxvirus expressing GM-CSF. Mol. Ther. *14*, 361–370.

70. Bristol, J.A., Zhu, M., Ji, H., Mina, M., Xie, Y., Clarke, L., Forry-Schaudies, S., and Ennist, D.L. (2003). In vitro and in vivo activities of an oncolytic adenoviral vector designed to express GM-CSF. Mol. Ther. *7*, 755–764.

71. Vidal-Vanaclocha, F., Fantuzzi, G., Mendoza, L., Fuentes, A.M., Anasagasti, M.J., Martín, J., Carrascal, T., Walsh, P., Reznikov, L.L., Kim, S.H., et al. (2000). IL-18 regulates IL-1beta-dependent hepatic melanoma metastasis via vascular cell adhesion molecule-1. Proc. Natl. Acad. Sci. USA *97*, 734–739.

72. Kim, H.J., Song, D.E., Lim, S.Y., Lee, S.H., Kang, J.L., Lee, S.J., Benveniste, E.N., and Choi, Y.H. (2011). Loss of the promyelocytic leukemia protein in gastric cancer: implications for IP-10 expression and tumor-infiltrating lymphocytes. PLoS ONE *6*, e26264.

73. Muthuswamy, R., Berk, E., Junecko, B.F., Zeh, H.J., Zureikat, A.H., Normolle, D., Luong, T.M., Reinhart, T.A., Bartlett, D.L., and Kalinski, P. (2012). NF-κB hyperactivation in tumor tissues allows tumor-selective reprogramming of the chemokine microenvironment to enhance the recruitment of cytolytic T effector cells. Cancer Res. *72*, 3735–3743.

74. Nguyen, T.L., Makhlouf, N.T., Anthony, B.A., Teague, R.M., and DiPaolo, R.J. (2014). In vitro induced regulatory T cells are unique from endogenous regulatory T cells and effective at suppressing late stages of ongoing autoimmunity. PLoS ONE *9*, e104698.

75. Kupz, A., Scott, T.A., Belz, G.T., Andrews, D.M., Greyer, M., Lew, A.M., Brooks, A.G., Smyth, M.J., Curtiss, R., 3rd, Bedoui, S., and Strugnell, R.A. (2013). Contribution of Thy1+ NK cells to protective IFN-γ production during Salmonella typhimurium infections. Proc. Natl. Acad. Sci. USA *110*, 2252–2257.

76. Gillard, G.O., Bivas-Benita, M., Hovav, A.H., Grandpre, L.E., Panas, M.W., Seaman, M.S., Haynes, B.F., and Letvin, N.L. (2011). Thy1+ NK [corrected] cells from vaccinia virus-primed mice confer protection against vaccinia virus challenge in the absence of adaptive lymphocytes. PLoS Pathog. *7*, e1002141.

77. Herberman, R.B., Nunn, M.E., and Holden, H.T. (1978). Low density of Thy 1 antigen on mouse effector cells mediating natural cytotoxicity against tumor cells. J. Immunol. *121*, 304–309.

78. DiNapoli, J.M., Kotelkin, A., Yang, L., Elankumaran, S., Murphy, B.R., Samal, S.K., Collins, P.L., and Bukreyev, A. (2007). Newcastle disease virus, a host range-restricted virus, as a vaccine vector for intranasal immunization against emerging pathogens. Proc. Natl. Acad. Sci. USA *104*, 9788–9793.

79. Lichty, B.D., Power, A.T., Stojdl, D.F., and Bell, J.C. (2004). Vesicular stomatitis virus: re-inventing the bullet. Trends Mol. Med. *10*, 210–216.

80. Zamarin, D., and Palese, P. (2012). Oncolytic Newcastle disease virus for cancer therapy: old challenges and new directions. Future Microbiol. *7*, 347–367.

81. Lee, C.K., Rao, D.T., Gertner, R., Gimeno, R., Frey, A.B., and Levy, D.E. (2000). Distinct requirements for IFNs and STAT1 in NK cell function. J. Immunol. *165*, 3571–3577.

82. Nguyen, K.B., Salazar-Mather, T.P., Dalod, M.Y., Van Deusen, J.B., Wei, X.Q., Liew, F.Y., Caligiuri, M.A., Durbin, J.E., and Biron, C.A. (2002). Coordinated and distinct roles for IFN-alpha beta, IL-12, and IL-15 regulation of NK cell responses to viral infection. J. Immunol. *169*, 4279–4287.

83. Lucas, M., Schachterle, W., Oberle, K., Aichele, P., and Diefenbach, A. (2007). Dendritic cells prime natural killer cells by trans-presenting interleukin 15. Immunity *26*, 503–517.

84. Sun, J.C., and Lanier, L.L. (2011). NK cell development, homeostasis and function: parallels with $CD8^+$ T cells. Nat. Rev. Immunol. *11*, 645–657.

85. Lodolce, J.P., Boone, D.L., Chai, S., Swain, R.E., Dassopoulos, T., Trettin, S., and Ma, A. (1998). IL-15 receptor maintains lymphoid homeostasis by supporting lymphocyte homing and proliferation. Immunity *9*, 669–676.

86. Verbist, K.C., and Klonowski, K.D. (2012). Functions of IL-15 in anti-viral immunity: multiplicity and variety. Cytokine *59*, 467–478.

87. Pol, J.G., Zhang, L., Bridle, B.W., Stephenson, K.B., Rességuier, J., Hanson, S., Chen, L., Kazdhan, N., Bramson, J.L., Stojdl, D.F., et al. (2014). Maraba virus as a potent oncolytic vaccine vector. Mol. Ther. *22*, 420–429.

88. Ockert, D., Schirrmacher, V., Beck, N., Stoelben, E., Ahlert, T., Flechtenmacher, J., Hagmüller, E., Buchcik, R., Nagel, M., and Saeger, H.D. (1996). Newcastle disease virus-infected intact autologous tumor cell vaccine for adjuvant active specific immunotherapy of resected colorectal carcinoma. Clin. Cancer Res. *2*, 21–28.

89. Boyman, O., Cho, J.H., and Sprent, J. (2010). The role of interleukin-2 in memory CD8 cell differentiation. Adv. Exp. Med. Biol. *684*, 28–41.

90. Seymour, L.W., and Fisher, K.D. (2016). Oncolytic viruses: finally delivering. Br. J. Cancer *114*, 357–361.

91. Xin, Y., Yin, M., Zhao, L., Meng, F., and Luo, L. (2017). Recent progress on nanoparticle-based drug delivery systems for cancer therapy. Cancer Biol. Med. *14*, 228–241.

92. Marcu, A., Pop, S., Dumitrache, F., Mocanu, M., Niculite, C.M., Gherghiceanu, M., Lungu, C.P., Fleaca, C., Ianchis, R., Barbut, A., et al. (2013). Magnetic iron oxide nanoparticles as drug delivery system in breast cancer. Appl. Surf. Sci. *281*, 60–65.

93. Santry, L.A., McAusland, T.M., Susta, L., Wood, G.A., Major, P.P., Petrik, J.J., Bridle, B.W., and Wootton, S.K. (2017). Production and Purification of High-Titer Newcastle Disease Virus for Use in Preclinical Mouse Models of Cancer. Mol. Ther. Methods Clin. Dev. *9*, 181–191.

94. Ramakrishnan, M.A. (2016). Determination of 50% endpoint titer using a simple formula. World J. Virol. *5*, 85–86.

OMTO, Volume *20*

Supplemental Information

Combining vanadyl sulfate with Newcastle disease virus potentiates rapid innate immune-mediated regression with curative potential in murine cancer models

Thomas M. McAusland, Jacob P. van Vloten, Lisa A. Santry, Matthew M. Guilleman, Amira D. Rghei, Edgar M. Ferreira, Joelle C. Ingrao, Rozanne Arulanandam, Pierre P. Major, Leonardo Susta, Khalil Karimi, Jean-Simon Diallo, Byram W. Bridle, and Sarah K. Wootton

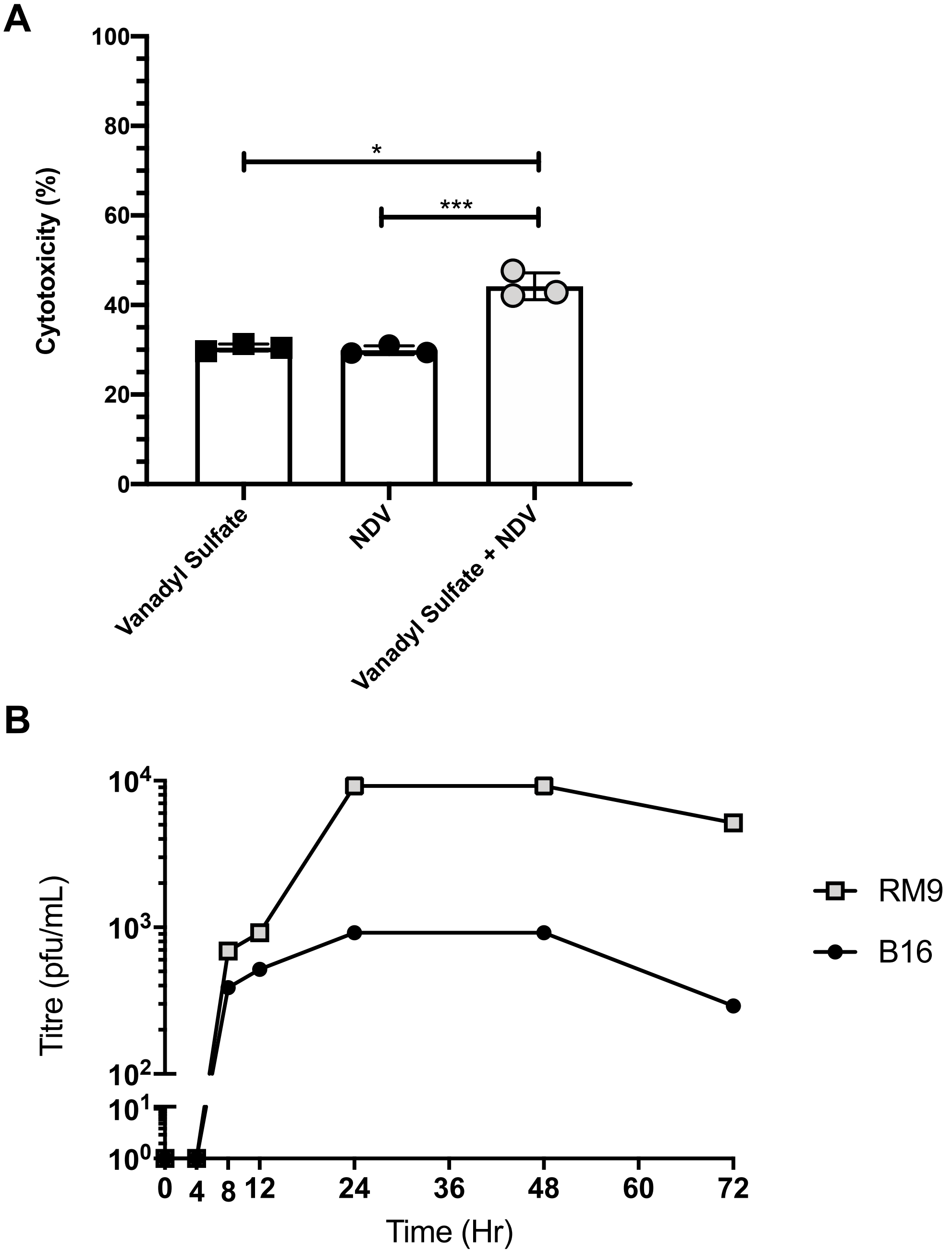

Supplementary Figure 1. (A) Quantification of lactate dehydrogenase (LDH) release from B16-F10 cells treated with NDV, vanadyl sulfate (VS) or a combination of NDV + VS. B16-F10 cells were plated in a 96 well plate at a density of $1x10^4$ cells per well. NDV was administered at an MOI of 0.01 and vanadyl sulfate at a concentration of 50 μM. 48 hours after administration of therapies, cell death was quantified by measuring the amount of LDH released from dying cells using the Pierce LDH cytotoxicity assay kit (cat no. 88953 ThermoFisher). Cytotoxicity was measured by subtracting LDH content in remaining viable cells from total LDH in untreated controls. Significance was determined using a one-way ANOVA test (*p<0.05, ***p<0.001). **(B)** Growth curve of NDV-GFP-F3aa-L289A in murine cancer cell lines. NDV was administered at an MOI of 0.5 to B16-F10 or RM9 cancer cells and supernatant was collected at 0, 4, 8, 12, 24, 48, and 72 hours post infection and applied to newly seeded chicken DF-1 cells to determine titre in pfu/mL. Titers represent an average of triplicates for each time point of each cell line.

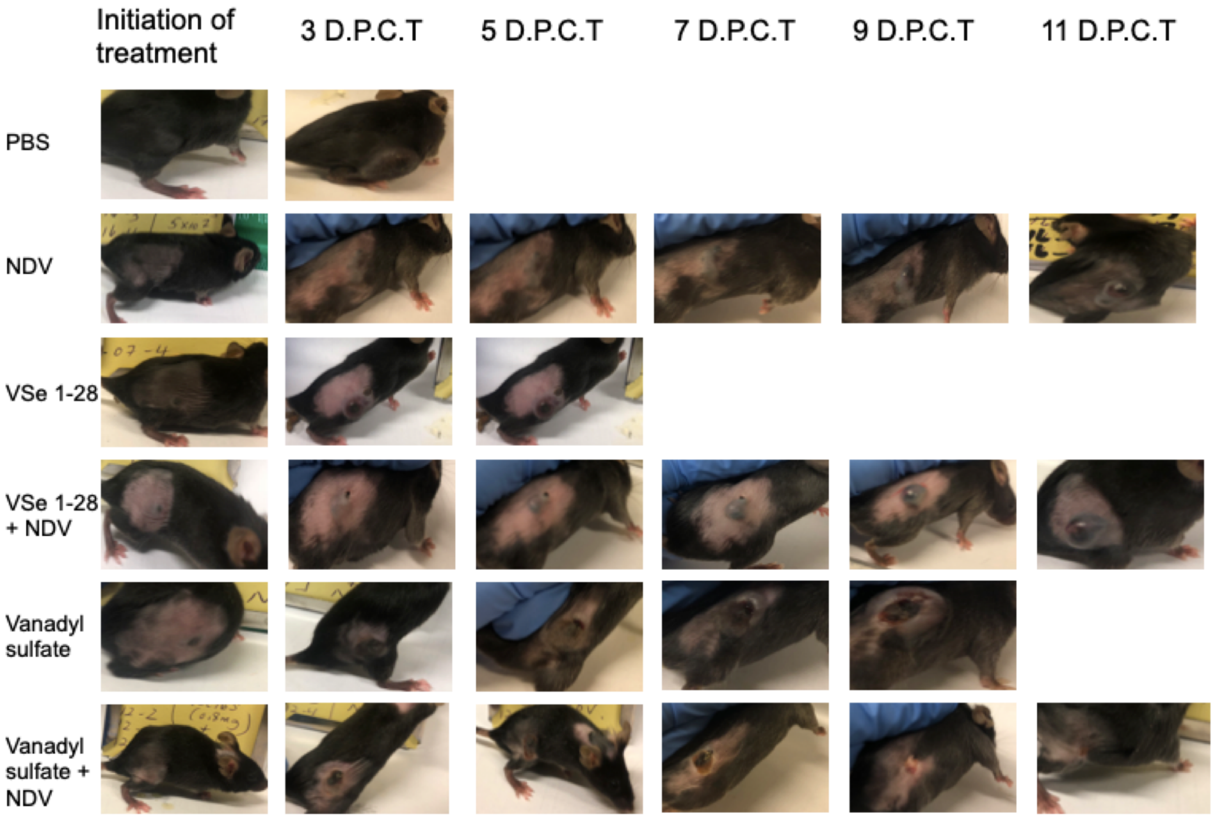

Supplementary Figure 2. Effect of combination therapies on survival in the B16-F10 murine melanoma model. B16-F10 cells ($5x10^5$ cells) were implanted intradermally and when tumors reached 5 mm in any direction, therapies (phosphate buffered saline (PBS), vanadyl sulfate, VSe 1-28, NDV, or a combination of drug plus NDV) were administered intratumorally every other day for a total of three treatments and tumor volume monitored,. Vanadyl sulfate (40 mg/kg) or VSe1-28 (40 mg/kg) were administered in a volume of 20 uL followed 4 hours later with an intratumoral injection of 5x107 pfu/mL NDV. A representative mouse from each treatment group was imaged starting from the initiation of treatment and varying days post cessation of treatment (D.P.C.T), lack of figure indicates endpoint reached.

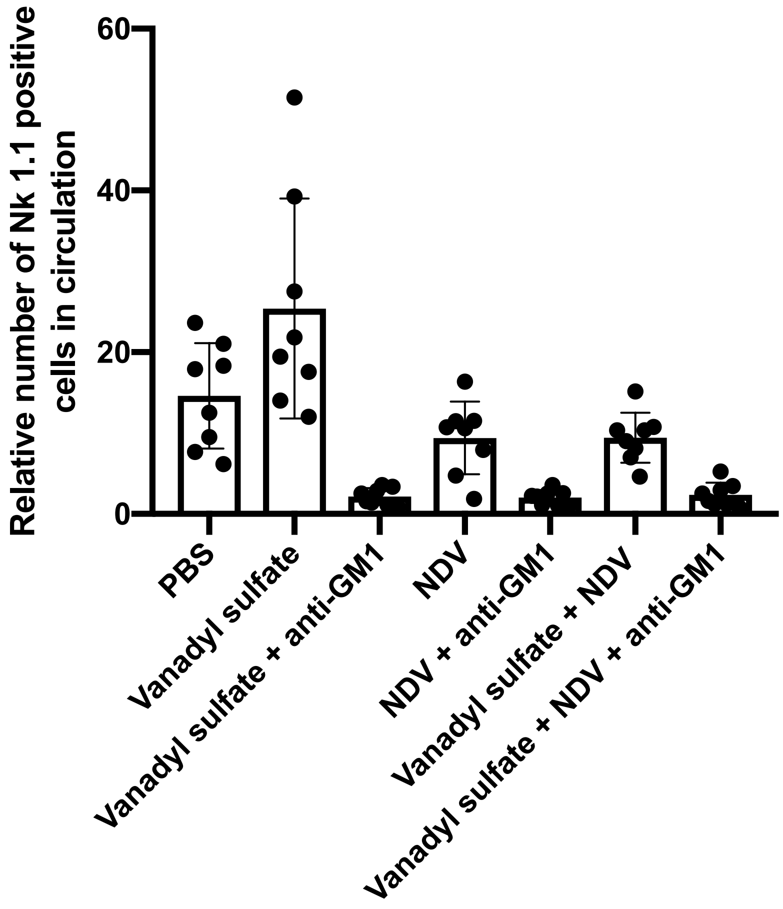

Supplementary Figure 3. Confirmation of NK cell depletion of mice used in vanadyl sulfate plus NDV and NK depletion survival. Blood was drawn retro-orbitally from mice 36 hours after a single treatment of PBS, vanadyl sulfate, NDV, or vanadyl sulfate plus NDV, or the same treatments used in combination with anti-Asialo-GM1 antibody. Blood was then processed using the following antibodies for flow cytometric analysis: BV 421anti-mouse CD3 (BioLegend, cat no. 100336, clone 145-2C11, San Diego, CA), FITC anti-mouse CD69 (BD Bioscience, cat no. 553236, clone H1.2F3, San Jose, CA), APC anti-mouse NK1.1 (BD Bioscience, cat no. 550627, clone PK136, San Jose, CA), and Fc Block-CD16/32 (BioLegend, cat no. 101320, clone 93, San Diego, CA). Cells were isolated from blood, and processed for surface labelling with antibodies against CD3, and NK 1.1, in a single staining panel, followed by the application of fixable viability dye Zombie NIR (BioLegend, San Diego, CA) to label dead cells, followed by fixation with IC fixation buffer (eBioscience, cat no. 00-8222-49, San Diego, CA). Data were acquired using a FACS Canto II flow cytometer with FACSDiva version 8.0 software (BD Biosciences, San Jose, CA) and analyzed using FlowJo version 10.6.2 software (BD Biosciences, San Jose, CA).

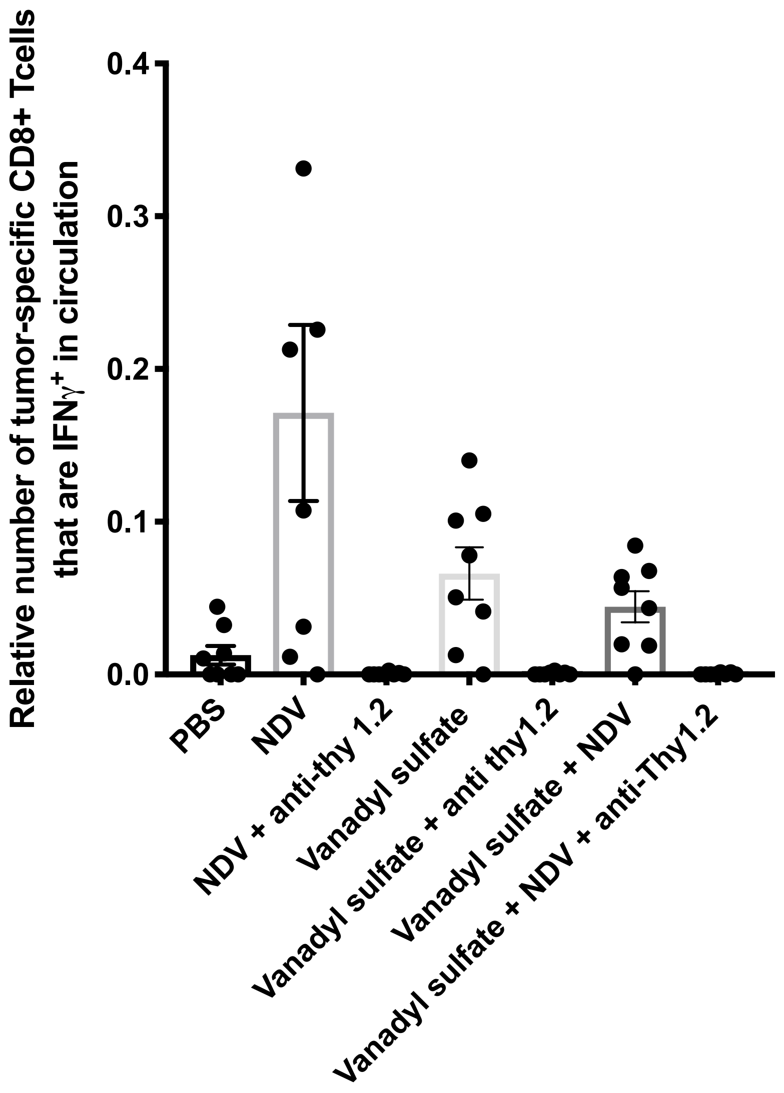

Supplementary Figure 4. Confirmation of T-cell depletion of mice used in vanadyl sulfate plus NDV and T-cell depletion survival. B16-F10 cells seeded at a density of 1×10^5 cells per well in a round bottom 96-well plate was stimulated with 50 units of murine IFN-γ recombinant protein (eBioscience, cat no. 14-8311-63, San Diego, CA) for 48 hours. Blood was drawn 10 days after the first dose of PBS, vanadyl sulfate (40 mg/kg), NDV (5×10^7 pfu/mL), or a combination of vanadyl sulfate plus NDV, or the same treatments used in combination with anti-mouse Thy1.2 (CD90.2; cat no. BE0076, clone 30H12; Bioxcell, Lebanon, NH) antibody and applied to IFN-γ -stimulated B16-F10. Cells were then treated with Fc Block-CD16/32 (BioLegend, cat no. 101320, clone 93, San Diego, CA) , stained and analyzed by flow cytometry after surface staining with antibodies against CD3, FITC anti-mouse CD4 (eBioscience, cat no. 11-0043-85, clone RM4-4, San Diego, CA) and BV 510 anti-mouse CD8 (BioLegend, cat no. 100752, clone 53-6.7, San Diego, CA), and intracellular staining against IFN-γ. T-cell populations were assessed to confirm complete depletion for survival study. Data were acquired using a FACS Canto II flow cytometer with FACSDiva version 8.0 software (BD Biosciences, San Jose, CA) and analyzed using FlowJo version 10.6.2 software (BD Biosciences, San Jose, CA).

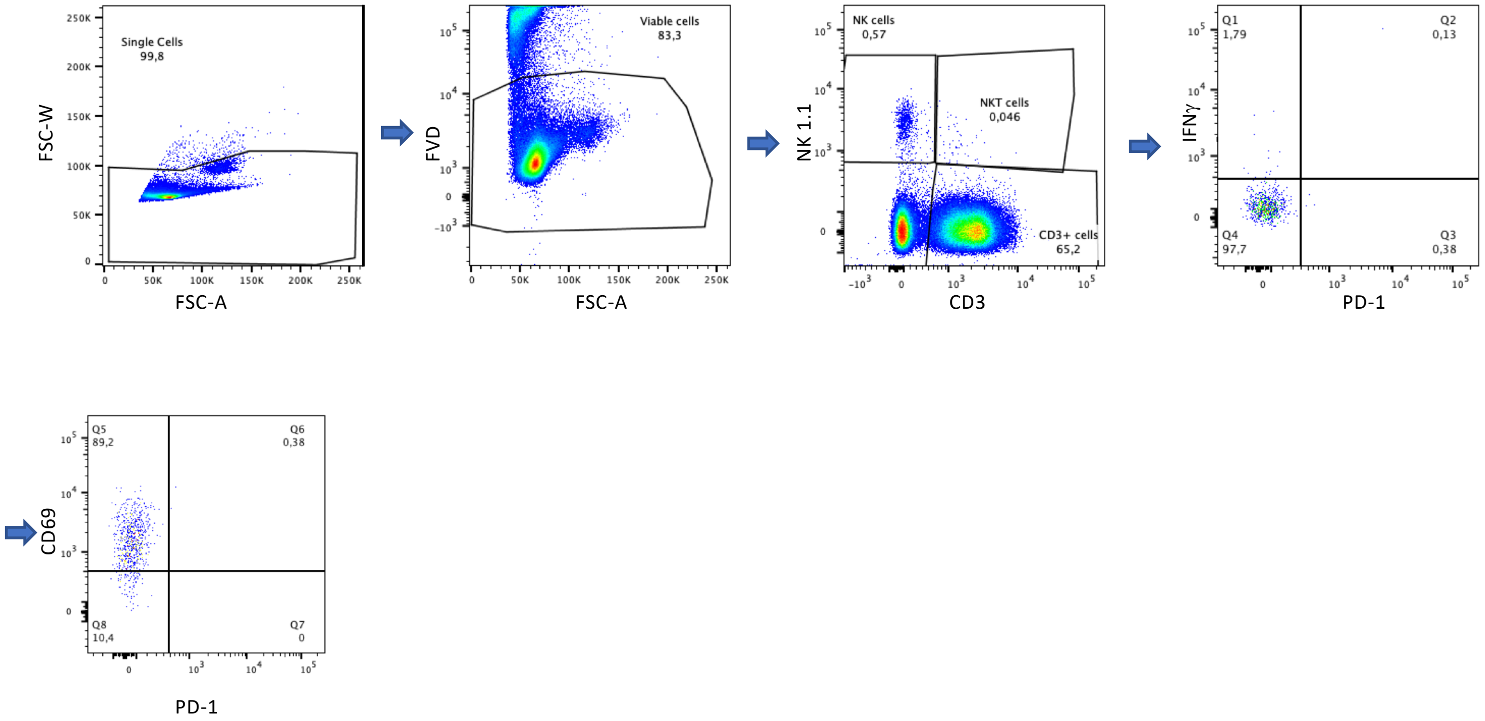

Supplementary Figure 5. Gating strategy to identify IFNγ producing NK cells in the tumor draining lymph node. Gating strategy used to identify single cells, viable cells and NK cells (NK 1.1+ CD3-). Additionally, IFNγ producing NK cells, followed by CD69+ NK cells were identified. Tumor draining lymph nodes were obtained from B16-F10 tumor bearing mice 36 hours after being treated with PBS, vanadyl sulfate, NDV, or a combination of vanadyl sulfate and NDV. Data were acquired using a FACS Canto II flow cytometer with FACSDiva version 8.0 software (BD Biosciences, San Jose, CA) and analyzed using FlowJo version 10.6.2 software (BD Biosciences, San Jose, CA).

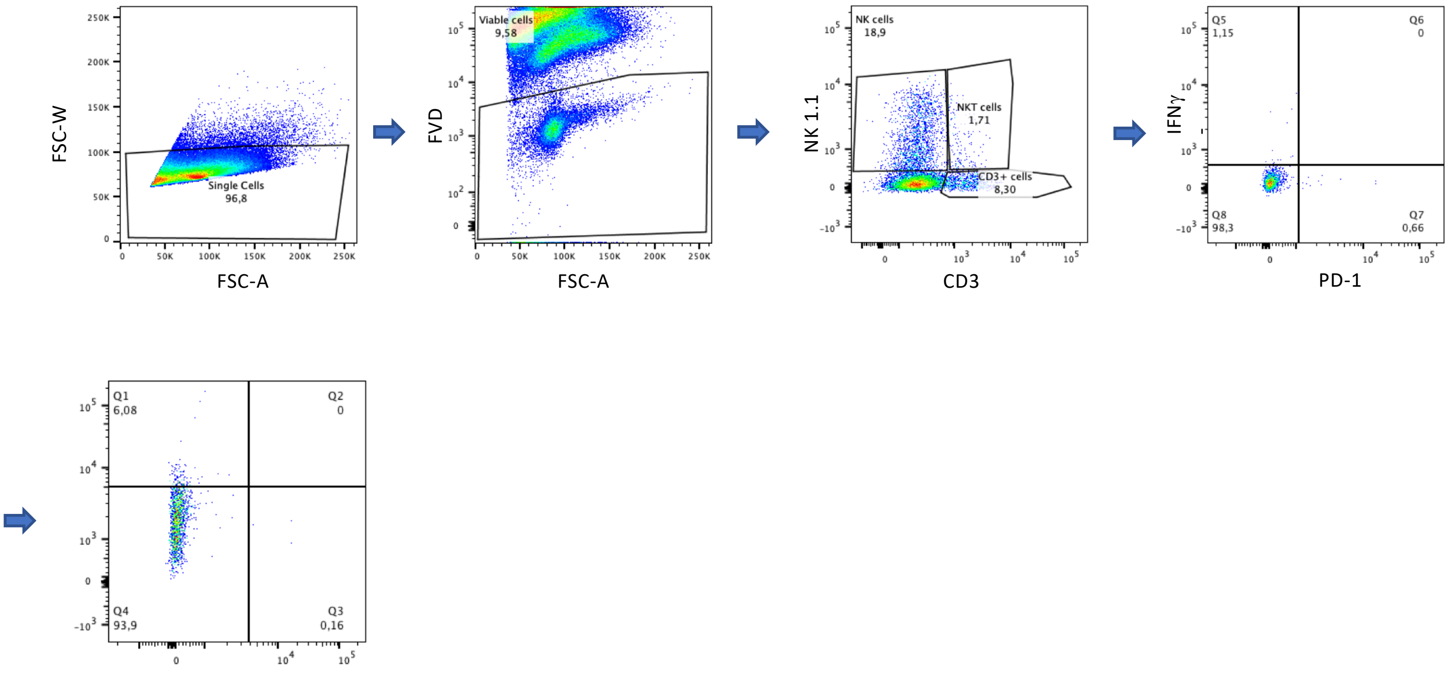

Supplementary Figure 6. Gating strategy to identify IFNγ producing NK cells in the tumor micro-environment. Gating strategy used to identify single cells, viable cells and NK cells (NK 1.1^{+} CD3^{-}). Additionally, IFNγ producing NK cells, followed by CD69+ NK cells were identified. Tumours were obtained from B16-F10 tumor bearing mice 36 hours after being treated with PBS, vanadyl sulfate, NDV, or a combination of vanadyl sulfate and NDV. Data were acquired using a FACS Canto II flow cytometer with FACSDiva version 8.0 software (BD Biosciences, San Jose, CA) and analyzed using FlowJo version 10.6.2 software (BD Biosciences, San Jose, CA).

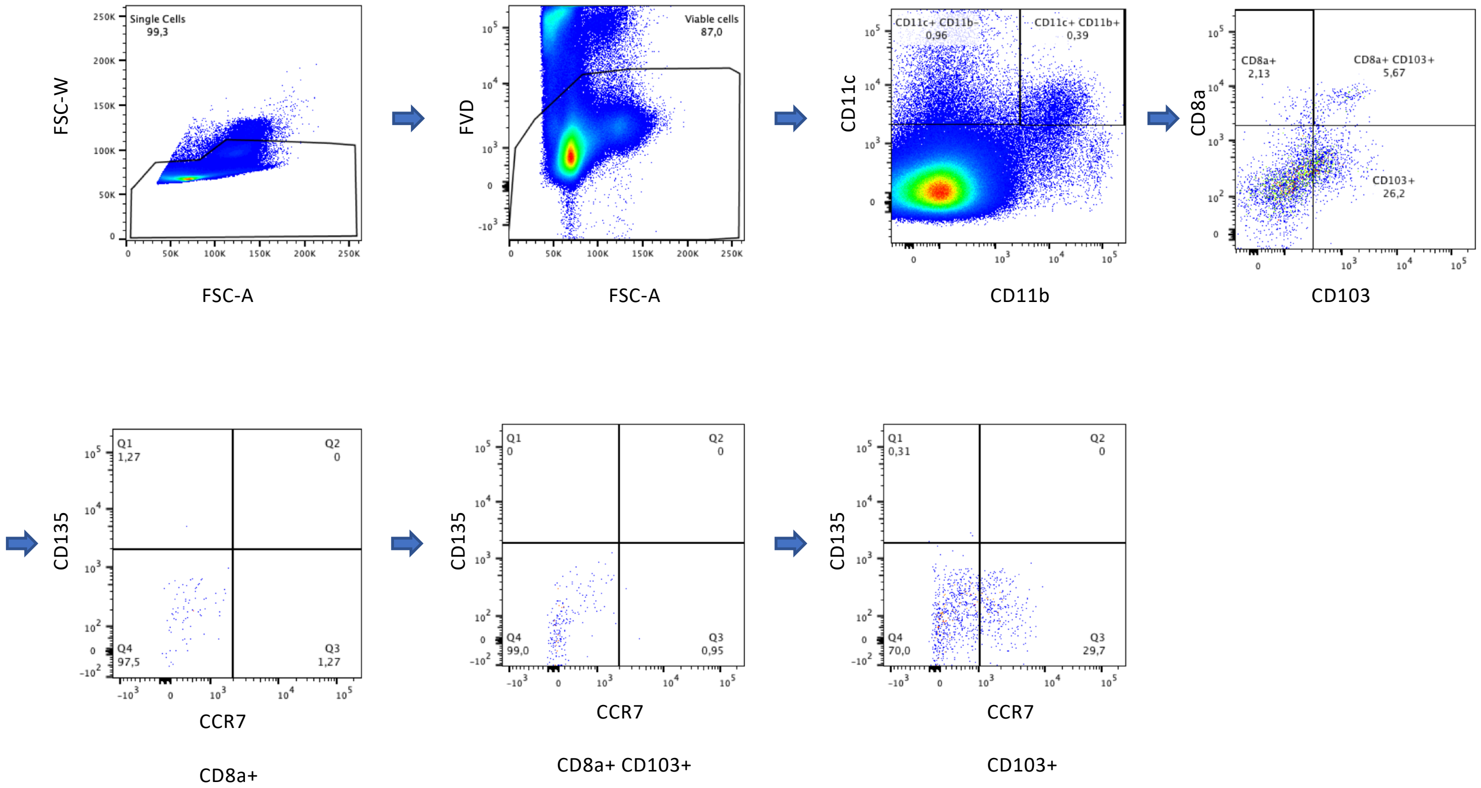

Supplementary Figure 7. Gating strategy to identify classical dendritic cell (cDC) populations in the tumor draining lymph node. Gating strategy used to identify single cells, viable cells, and then divided cells into CD11b+ and CD11c+ cells. Cells were then further divided using CD8a and CD103 staining, which identified single and double positives. CD8a+, CD103+, or CD8a and CD103+ populations were then analyzed for CD135 and CCR7. Tumours were obtained from B16-F10 tumor bearing mice 36 hours after being treated with PBS, vanadyl sulfate, NDV, or a combination of vanadyl sulfate and NDV. Data were acquired using a FACS Canto II flow cytometer with FACSDiva version 8.0 software (BD Biosciences, San Jose, CA) and analyzed using FlowJo version 10.6.2 software (BD Biosciences, San Jose, CA).

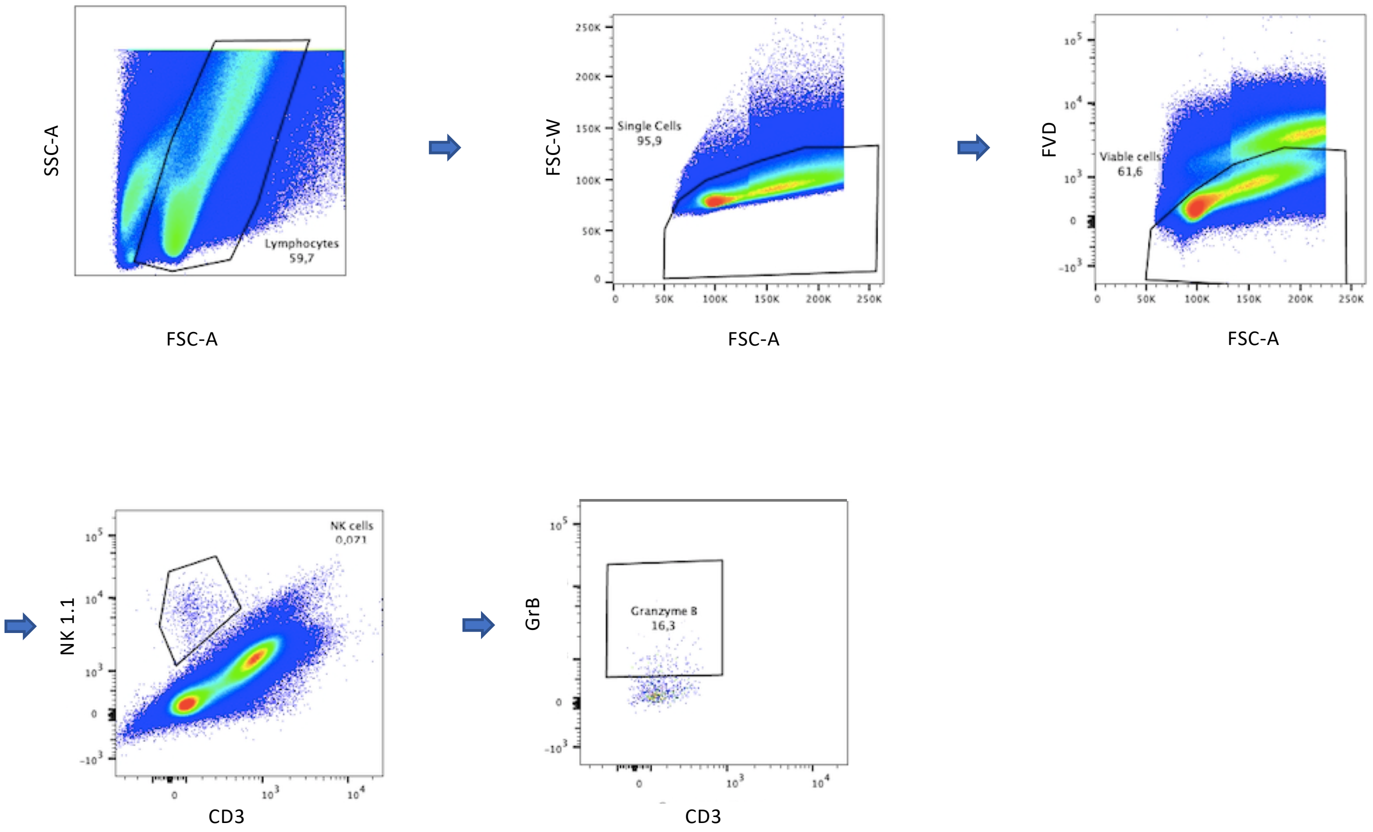

Supplementary Figure 8. Gating strategy to identify Granzyme-B producing NK cells at an acute time point in the tumor micro-environment. Gating strategy used to identify lymphocytes, single cells, viable cells, and NK 1.1+ CD3- cells, as NK cells. NK cells were then analyzed for Granzyme-B levels. Tumours were obtained from B16-F10 tumor bearing mice 24 hours after being treated with PBS, vanadyl sulfate, NDV, or a combination of vanadyl sulfate and NDV. Data were acquired using a FACS Canto II flow cytometer with FACSDiva version 8.0 software (BD Biosciences, San Jose, CA) and analyzed using FlowJo version 10.6.2 software (BD Biosciences, San Jose, CA).

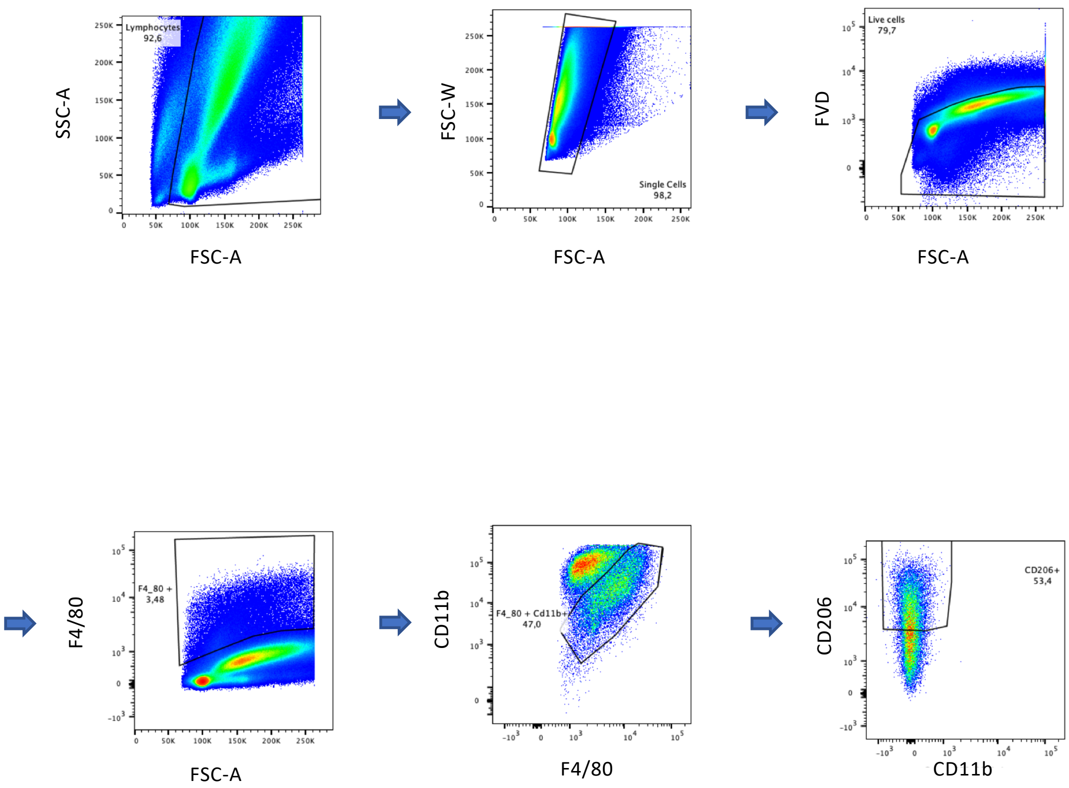

Supplementary Figure 9. Gating strategy to identify CD206 positive macrophage populations. Gating strategy used to identify lymphocytes, single cells, viable cells followed by F4/80+ cells. Cells were then identified as CD11b+ and lastly CD206+. Tumours were obtained from B16-F10 tumor bearing mice 24 hours after being treated with PBS, vanadyl sulfate, NDV, or a combination of vanadyl sulfate and NDV. Data were acquired using a FACS Canto II flow cytometer with FACSDiva version 8.0 software (BD Biosciences, San Jose, CA) and analyzed using FlowJo version 10.6.2 software (BD Biosciences, San Jose, CA).
